# European data sources for computing burden of (potential) vaccine-preventable diseases in ageing adults

**DOI:** 10.1186/s12879-021-06017-7

**Published:** 2021-04-13

**Authors:** Estelle Méroc, Janeri Fröberg, Timea Almasi, Brita Askeland Winje, Alejandro Orrico-Sánchez, Anneke Steens, Scott A. McDonald, Kaatje Bollaerts, Mirjam J. Knol

**Affiliations:** 1 P95 Epidemiology and Pharmacovigilance, Koning Leopold III laan 1, 3001 Leuven, Belgium; 2 Radboud University Medical Center, Radboud Institute for Molecular Life Sciences, Laboratory of Medical Immunology, Section Paediatric Infectious Diseases, Geert Grooteplein 21, 6525 EZ Nijmegen, the Netherlands; 3 Syreon Research Institute, Mexikoi str. 65/A, Budapest, 1142 Hungary; 4 Department of Infection Control and Vaccine, Norwegian Institute of Public Health, PO Box 222, Skøyen, 0213 Oslo, Norway; 5 Vaccines Research Unit, FISABIO (the Valencia Foundation for the Promotion of Health and Biomedical Research), Av. Catalunya, 21, 46020 Valencia, Spain; 6 Centre for Infectious Disease Control, National Institute for Public Health and the Environment (RIVM), PO Box 1, 3720 BA Bilthoven, the Netherlands

**Keywords:** Infectious disease, Elderly, Burden of disease, Vaccine, Public health

## Abstract

**Background:**

To guide decision-making on immunisation programmes for ageing adults in Europe, one of the aims of the Vaccines and InfecTious diseases in the Ageing popuLation (IMI2-VITAL) project is to assess the burden of disease (BoD) of (potentially) vaccine-preventable diseases ((P)VPD). We aimed to identify the available data sources to calculate the BoD of (P)VPD in participating VITAL countries and to pinpoint data gaps. Based on epidemiological criteria and vaccine availability, we prioritized (P) VPD caused by Extra-intestinal pathogenic *Escherichia coli* (ExPEC), norovirus, respiratory syncytial virus, *Staphylococcus aureus*, and pneumococcal pneumonia.

**Methods:**

We conducted a survey on available data (e.g. incidence, mortality, disability-adjusted life years (DALY), quality-adjusted life years (QALY), sequelae, antimicrobial resistance (AMR), etc.) among national experts from European countries, and carried out five pathogen-specific literature reviews by searching MEDLINE for peer-reviewed publications published between 2009 and 2019.

**Results:**

Morbidity and mortality data were generally available for all five diseases, while summary BoD estimates were mostly lacking. Available data were not always stratified by age and risk group, which is especially important when calculating BoD for ageing adults. AMR data were available in several countries for *S. aureus* and ExPEC.

**Conclusion:**

This study provides an exhaustive overview of the available data sources and data gaps for the estimation of BoD of five (P) VPD in ageing adults in the EU/EAA, which is useful to guide pathogen-specific BoD studies and contribute to calculation of (P)VPDs BoD.

**Supplementary Information:**

The online version contains supplementary material available at 10.1186/s12879-021-06017-7.

## Background

In 2019, ageing adults (≥50 years) represented about 40% of the population in the European Union (EU), and this proportion is expected to reach 50% by 2025 [1–5]. This rising demographic trend is expected to lead to a growing demand for healthcare as infectious diseases in ageing adults, especially those affecting the elderly (≥65 years), already place a significant health and economic burden on individuals and societies [1, 6, 7]. Ageing adults experience a natural decline in their immune response, also called immuno-senescence [5, 6], increasing their risk of contracting infectious diseases, as well as their risk of a severe disease course. Ageing patients often present with atypical symptoms, thereby complicating the clinical diagnosis [8–10]. Moreover, for persons living in elderly care institutions, close living arrangements can further increase the risk of infection.

Prevention strategies, and more specifically vaccination, are often a cost-effective way to decrease the incidence of infectious diseases, thereby contributing to healthy ageing [11–13]. Although effective vaccines are already available to prevent some major infectious diseases in ageing adults, such as influenza and pneumococcal disease [1, 14], their vaccination coverage rates remain relatively low in Europe [6]. This is likely due to limited awareness of the adverse impacts on health arising from infectious diseases, and existing gaps in the promotion of adult vaccinations by public health authorities [1, 6]. To guide decision-making about potential prevention strategies for this age-group, it is essential to understand the burden of disease (BoD) and to identify existing data gaps.

In this context, the second Innovative Medicine Initiative (IMI2) supported the Vaccines and InfecTious Diseases in the Ageing PopuLation (IMI2-VITAL) project [15], which aims to address the current challenges of (potentially) vaccine-preventable diseases ((P)VPD) in ageing adults in the EU/EEA. The project will provide evidence-based knowledge on possible specific vaccination strategies to enhance healthy ageing.

We present here a comprehensive review of currently available data sources in the EU/EEA for 2009–2019 to calculate the BoD of (P)VPD in ageing adults and describe the data gaps.

## Methods

### Disease selection

We identified (P)VPD based on WHO summary information [16] and input from project members. We subsequently selected diseases to review based on their relevance for EU/EEA and the ageing adult population, and the availability of a vaccine in at least phase 2 of clinical development. This resulted in a list of seven infectious diseases: influenza, varicella/herpes zoster, herpes simplex virus, norovirus, respiratory syncytial virus (RSV), *Staphylococcus aureus* (*S. aureus*), extra-intestinal pathogenic *Escherichia coli* (ExPEC), and pneumococcal pneumonia (PnPn). This list was further narrowed down to diseases with a high BoD and/or lack of BoD data. The ranking resulted in five (P)VPD to be assessed on data sources for this review: norovirus, *S. aureus*, ExPEC, PnPn, and RSV.

### National disease expert surveys

An online survey on BoD data was developed for each of the selected (P)VPD and sent to disease experts from IMI2-VITAL participating countries (Belgium, Denmark, Hungary, Italy, The Netherlands, Norway and Spain). The experts were identified by the IMI2-VITAL partners who aimed to adhere to the European Centre for Disease Prevention and Control (ECDC) national focal points. The parameters of interest included summary measures (disability-adjusted life year (DALY), quality-adjusted life year (QALY)), utility/disability, morbidity, mortality, antimicrobial resistance (AMR), other pathogen-associated and patient registry data.

The surveys, developed in Google Forms, were based on case definitions described in Table 1 with a supplementary example available in Additional file 1. The survey responses were summarised by listing the available data for each BoD indicator by country.

**Table 1:** case definitions used for the surveys of national disease experts

Pathogen	Disease and case definition
Extra-intestinal pathogenic *Escherichia coli* (ExPEC)	Invasive ExPEC disease: laboratory-confirmed sepsis-causing ExPEC, based on invasive isolates from blood or cerebrospinal fluid
*Norovirus* *(NoV)*	1. Norovirus infection: diarrhoea or vomiting or both in a 24-h period confirmed by a positive laboratory test (nucleic acid amplification assay or antigen detection or electron microscopy) or 2. Acute gastroenteritis: diarrhoea or vomiting or both in a 24-h period without laboratory confirmation.
*Pneumococcal pneumonia* (*PnPn)*	1. Non-bacteraemic PnPn: clinical symptoms consistent with pneumonia, with a positive pneumococcal urinary antigen test (UAT) but a negative blood culture, or 2. All-cause pneumonia: clinical symptoms consistent with pneumonia, with or without x-ray confirmation, without bacteriologic confirmation
*Respiratory Syncytial Virus* *(RSV)*	1. Community based Acute Infection (ARI caused by RSV): sudden onset of at least one of the following symptoms: shortness of breath; cough; sore throat and coryza and a laboratory confirmation with RSV, or 2. Hospital-based Extended Severe Acute RSV Infection (SARI caused by RSV): abovementioned symptoms with onset within the last ten days, requiring hospitalisation and a laboratory confirmation with RSV. This could include severe lower respiratory tract infections, like pneumonia.
*Staphylococcus aureus* *(S. aureus)*	Invasive *S. aureus* infection: clinical symptoms consistent with bacteraemia or sepsis, and isolation of *S. aureus* from the blood or other sterile site (synovial fluid, cerebrospinal fluid, pleural fluid, bronchoalveolar lavage, or from a sterile taken deep-seated abscess).

### Literature reviews

A literature review was conducted by searching the MEDLINE database (via PubMed) for peer-reviewed publications with data relevant to BoD calculations. A search strategy was developed for each disease, consisting of a common part across all diseases and a disease-specific part (Additional file 2). The former contained search terms on BoD parameters, age group and the period of interest, while the latter contained the name of the pathogen, clinical syndromes and related synonyms. The BoD parameters included summary measures (DALY, QALY, years of life lost (YLL), years lived with disability (YLD) and Quality of Life (QoL)), disability/utility, morbidity mortality and AMR data. The period of interest was restricted to the last 10 years (01/01/2009–23/05/2019), except for PnPn, where only publications since 01/01/2012 were included. The rationale was to exclude data reported before large-scale paediatric use of more-valent pneumococcal conjugate vaccines (PCV10/PCV13), as this has impacted the BoD in the ageing population due to indirect effects [17, 18].

The literature selection process and the number of excluded publications by reason were documented and presented in PRISMA flow-diagrams (Additional file 3). Titles and abstracts of identified records were screened by one reviewer to identify studies that fulfilled the selection criteria. Studies were excluded if there was 1) No English abstract and an irrelevant title, 2) No human subjects, 3) No relation to the disease of interest, 4) No new evidence described (editorials, letters, case reports/series), 5) No relevant data (this criterion was further specified in each disease area according to the characteristics of the disease in question, see definitions on the PRISMA flowcharts), 6) Only children, or 7) If the study was conducted outside of Europe. A sample of 100 abstracts per disease was double-screened, and discrepancies were discussed among reviewers. Data from the selected publications were then extracted using a uniform Excel table for all diseases. Based on the available literature, summary tables were created to list the countries for which BoD parameters were available.

## Results

### Extra-intestinal pathogenic *Escherichia coli*

#### Survey

The Netherlands, Denmark and Norway use the proposed case definition in their surveillance (Table 1). In Italy, only a subset of laboratories within the European AMR surveillance network (EARS-net) use this case definition. Hungary uses a non-agent specific definition for Hospital-Associated Bloodstream Infections (HA-BSI) [19], Belgium and Spain do not collect ExPEC-specific data. None of the countries reported summary BoD measures (Table 2). Stratification by age-group and risk factors was reported as possible in the six countries for which morbidity data are available (Hungary, Denmark, Norway, Italy, and The Netherlands). ExPEC-mortality and sequelae data were rarely available. The latter could only be retrieved in The Netherlands. All countries collected AMR data.

**Table 2:** Data sources available to capture (P) VPD burden of disease (BoD) based on national disease expert surveys: Countries with available data sources

Indicator	ExPEC	NoV	PnPn	RSV	*S.aureus*
Summary BoD					
DALY	not available	BE, NL, DK	NL	BE, DK	BE
QALY	not available	not available	BE, NL	not available	not available
Disability/utility					
utility weights	not available	not available	NL	not available	not available
disability weights	not available	not available	not available	not available	not available
Morbidity					
incidence/prevalence	HU, DK, IT, NO, NL	BE, DK, ES, HU, IT, NL	BE, ES, HU, IT NL, NO	BE, NL, ES, DK, IT	BE, HU, NL, DK, IT
serotype data	NA	NA	ES, IT	NA	NA
attributable fraction	NA	NA	BE, NL	NA	NA
sequelae	NL	not available	not available	DK	NL, ES, DK
disease duration	DK, NO	ES, NL	not available	BE, ES, DK	ES
hospitalization need	HU, DK, IT, NO	BE, ES, HU, NL	BE, HU, NL	BE, NL, ES, DK	BE, HU, ES
hospitalization duration	HU, DK, IT, NO	BE, ES, HU, NL	BE, NL	BE, NL, ES, DK	BE, HU, ES
stratification age/RF	HU, IT, NL, NO	DK, HU	BE, ES, HU, IT, NL, NO	BE, NL, ES, DK	BE, HU, ES, NL, IT
completeness assessment	HU	BE, NL	BE, NL	NL	HU, ES, DK
Mortality					
mortality	NO, IT	BE, DK, ES, HU	BE, ES, HU, IT, NL	NL, BE, ES, DK	ES
serotype data	NA	NA	NA	NA	DK
case-fatality rate	not available	not available	BE, HU	BE, ES, DK	DK
stratification age/RF	NO	HU	BE, ES, HU, IT, NL	BE, ES	
Antimicrobial resistance	DK, HU, IT, NL, NO	NA	NA	NA	BE, HU, NL, NO, ES, DK, IT
Other pathogen-associated data	DK, HU, NL, NO	NA	NA	NA	BE, HU, DK
Patient registry	NA	NA	NA	NL, ES	NA

Abbreviations: *NoV* norovirus, *PnPn* Pneumococcal pneumonia, *RSV* Respiratory Syncytial Virus, *S.aureus Staphylococcus aureus*, *ExPEC* Extra-intestinal pathogenic *Escherichia coli*, *BE* Belgium, *DK* Denmark, *ES* Spain, *HU* Hungary, *IT* Italy, *NL* the Netherlands, *NO* Norway; *NA* Not applicable

#### Literature review

A total of 940 publications were identified in the literature review, of which 41 were included for data extraction covering eleven countries (Additional files 3 and 4). Most publications came from the United Kingdom (UK) (*n* = 9), Denmark (*n* = 6) and Spain (*n* = 4) (Table 3). Additionally, seven publications included multiple countries for data extraction. Respectively, twelve and three publications focused on *E.coli* bacteraemia and urosepsis. Sixteen studies provided general Bloodstream Infection (BSI) morbidity data. Only eight studies presented *E.coli* bacteraemia incidence. Seven publications concerned BSI caused by AMR *E.coli* strains. Several countries presented data on *E.coli* BSI mortality (Denmark, Finland, Norway, Sweden and the UK) or AMR *E.coli*-related BSI mortality (Germany and Spain). A large European study reporting ICU-specific mortality data was also identified. Many publications focusing on AMR *E.coli* BSI were found. Two large AMR-focused surveillance systems were identified (SENTRY and EARS-Net) [20, 21]. Only one study reported DALYs for AMR *E.coli*, and no study reported disability/utility weights, disease duration, or the need for hospitalisation due to ExPEC (hospitalisation needs).

**Table 3:** Data sources available to capture (P) VPD based on literature reviews: Number of references and countries with available data sources

Indicator	ExPEC		NoV		PnPn		RSV		S.aureus	
	n	Countries	n	Countries	n	Countries	n	Countries	n	Countries
Summary BoD data	1	EU	3	NL, UK	2	NL	1	world	0	
Disability/utility	0		2	NL	2	CH, FR	1	world	0	
Morbidity										
incidence/prevalence	33	AT, CH, DE, EU, DK, ES, FI, FR, IT, NO, SE, UK	24	CH, DE, ES, FR, IT, NL, SE, UK	17	DE, ES, FI, FR, IT, PO, NL, SE	36	BE, CH, DE, EU, ES, FR, GR, HU, NL, PO, SE, UK, world	51	BA, CH, DE, DK, ES, EN, EU, FI, FR, GE, GR, IE, IS, IT, ME, MK, NL, NO, PO, RO, RS, SC, SE, TR, XK
NSS and AF^a^					14	BE, ES, IT, NL, SE, UK	11	BE, CH, EU, FR, UK, world		
sequelae	4	AT, CH, DE, DK, FR, UK	3	CH, NL		NA	2	FR, UK	30	AT, DE, DK, EN, ES, FI, FR, HR, IL, NO, RO, SC, SE
disease duration	0		8	DE, ES, FR, IT, NL, UK	4	BE, FR, NL, UK	1	world	3	DK, IL, SC
hospitalization need	0		14	CH, DE, FR, IT, NL, SE, UK	2	BE, FR	15	CH, ES, EU, FR, SE, UK, world	11	AT, DE, DK, HR, IS, IT, NO, SE
hospitalization duration	15	AT, DE, DK, EU, SE, UK	4	DE, NL, SE	13	ES, FR, IT, NL, UK	7	CH, ES, EU, FR	27	AT, BE, CH, DE, DK, ES, FI, FR, GR, HR, IE, NO, RO, SC, SE, TR
Age stratification	19	CH, DK, DE, EU, FI, FR, IT, NO, SE, UK	23	CH, DE, ES, FR, IT, NL, SE, UK	25	BE, ES, FI, FR, IT, NL, PO, SE	27	BE, CH, DE, ES, EU, FR, GR, HU, NL, PO, SE, UK, world	41	CH, DE, DK, EN, ES, EU, FI, IE, IS, IT, NO, SC, SE
Mortality										
pathogen-specific mortality	24	AT, DE, DK, ES, EU, FI, FR, NO, SE, UK	10	DE, NL, SE, UK					75	AT, BE, CH, DE, DK, EN, ES, FI, FR, HR, IE, IL, IS, NO, SC, SE, TR
case-fatality rate	5	EU, FI, NO, UK	2	DE, NL	13	DE, ES, FR, IT, NL, SE, UK	12	CH, ES, EU, FR, NL, SE, UK, world	10	DK, FI, FR, IL, NO, TR
Antimicrobial resistance	26	DE, DK, ES, EU, FI, FR, IT, UK		NA	1	PT		NA	63	AT, BA, BE, CH, DE, DK, EN, ES, EU, FI, FR, GE,GR,IE, IL, IS, IT, ME, MK, NL, NO, PO, PT, RO, RS, SC, SE,TR, XK

Abbreviations: *NoV* norovirus, *PnPn* Pneumococcal pneumonia, *RSV* Respiratory Syncytial Virus, *S.aureus* Staphylococcus aureus, *ExPEC* Extra-intestinal pathogenic Escherichia coli, *AT* Austria, *BA* Bosnia and Herzegovina, *BE* Belgium, *CH* Switzerland, *DE* Germany, *EN* England, *DK* Denmark, *ES* Spain, *EU* Europe, *FI* Finland, *FR* France, *GE* Georgia, *GR* Greece, *HR* Croatia, *IE* Ireland, *IL* Israel, *IS* Iceland, *IT* Italy, *MK* Macedonia, *NL* the Netherlands, *ME* Montenegro, *NO* Norway, *PO* Poland, *PT* Portugal, *RO* Romania, *RS* Serbia, *SC* Scotland, *SE* Sweden, *TR* Turkey, *UK* United Kingdom, *XK* Kosovo; ^a^NSS and AF: non-specific syndrome and attributable fraction

### Norovirus

#### Survey

Belgium, Italy and Spain use both the norovirus and syndromic (acute gastroenteritis) case definitions in their surveillance (Table 1). Denmark uses only the norovirus case definition. The Netherlands collects aggregated laboratory surveillance data on norovirus. Hungary and Norway collect data on norovirus outbreaks. The survey results indicated that the majority of countries collected norovirus morbidity data (6 out of 7), mortality data (4 out of 7), and hospitalisation data (4 out of 7) (Table 2). In addition, Belgium, Denmark and the Netherlands estimated DALYs specifically for norovirus.. However, only a few countries assessed the level of under-reporting or under-ascertainment (i.e. completeness) of morbidity data, and morbidity and mortality data were rarely stratified by age or risk factors. No country collected sequelae data.

#### Literature review

The literature review yielded 371 publications, of which 24 were selected for data extraction (Additional files 3 and 4). Data from eight countries were retrieved, mostly from the Netherlands (*n* = 6), the UK (*n* = 6) and Germany (*n* = 5) (Table 2). Four studies (from the UK and the Netherlands) estimated DALYs based on incidence data obtained via the population-based studies Sensor [22] and IID2 (Infectious Intestinal Disease in the community study) [23, 24]. Morbidity data were retrieved in 24 publications, half of them describing pathogen-specific data, in most cases stratified by age. Sequelae data were identified in three syndromic data studies. Norovirus and syndromic disease mortality data were identified in eight and three studies, respectively.

### Pneumococcal pneumonia

#### Survey

Spain and Italy use the non-bacteraemia PnPn case definition in their surveillance, whereas the other countries use only the all-cause pneumonia case definition (Table 1). Overall, PnPn-specific BoD data is lacking, as no country collected summary BoD data, and only Spain and Italy reported PnPn-morbidity and mortality data (Table 2). On the other hand, more data was available for all-cause pneumonia; Belgium and the Netherlands reported that DALY and/or QALYs have already been calculated or could be calculated for all-cause pneumonia. All-cause pneumonia incidence and hospitalisation data are available in most countries, and mortality data are available in the Netherlands, Belgium and Hungary.

#### Literature review

A total of 784 publications were identified, of which 40 were included for data extraction (Additional files 3 and 4). Most studies were conducted in Spain (*n* = 9), the Netherlands (*n* = 8), the UK (*n* = 7) and Italy (*n* = 5) (Table 2). Only two studies reported QALYs; both were based on the CAPITA study from the Netherlands on all-cause pneumonia [25, 26]. Two studies from France and Switzerland determined the QoL for all-cause pneumonia [27, 28]. For most studies, the incidence was based on hospital discharge data, either for all-cause pneumonia or PnPn. Incidence data on outpatients with all-cause pneumonia were only reported for France, the Netherlands and Belgium. Serotype-specific PnPn incidence data were available in three studies. Several studies reported the attributable fraction for pneumococci using hospital discharge data. Most studies reported age stratification data and half of them reported data for risk groups. Several studies reported mortality after PnPn or after all-cause pneumonia.

### Respiratory syncytial virus

#### Survey

Case definitions and methods to ascertain RSV cases differ widely by country (Table 1). In Belgium and Spain, laboratory testing is usually conducted only in a selection of hospitalized cases. In the Netherlands, while a subset of ARI cases undergoes laboratory testing for RSV and real-time SARI surveillance is being piloted, no official laboratory confirmation is needed for applying ICD-10 codes in hospitalized cases. Many hospitals in Norway conduct influenza like illness (ILI) surveillance with RSV and influenza panel testing, which results in RSV-positivity among ILI cases. Under-ascertainment and underreporting may bias the official frequency estimates as the completeness of reported frequency data are not assessed in any of the countries (Table 2). The only exception might be the Netherlands, where efforts have been made to assess data completeness for RSV cases identified in primary care. RSV sequelae data are available from Denmark, and are also currently being studied in the Netherlands, in the context of the RESCEU (Respiratory Syncytial virus Consortium in Europe) project [27]. Spain has reported that hospital records could be used as a possible source of sequelae data. As several countries use SARI case definitions for RSV (Belgium, Spain, the Netherlands, Denmark), there are available data on hospitalisations and RSV-specific mortality. In Belgium, sentinel hospitals report case-fatality and RSV-SARI mortality data, which can be stratified by age and risk groups. There are also case-fatality data in Spain, but only for younger age-groups and for a subset of cases which undergo laboratory testing.

#### Literature review

The literature search yielded a total of 711 publications, of which 46 were included in the data extraction (Additional files 3 and 4). The majority of studies originated from the UK (*n* = 12), followed by Sweden (*n* = 4), France (*n* = 3), Poland (*n* = 3), Spain (*n* = 3), Germany (*n* = 2), Greece (*n* = 2), the Netherlands (*n* = 2), Switzerland (*n* = 2), and Belgium (*n* = 1) (Table 2). In the literature review, the most widely used case definitions for sampling are based on ILI or ARI cases, or both. Although one article reported age-specific DALYs due to hospitalised lower or upper respiratory tract infections (LRTIs and URTIs) caused by RSV, no study described country-specific summary BoD estimates. In a study by Shi et al. [28], simple BoD data (RSV incidence, hospital admission rates, RSV positivity among hospitalised ARI cases and hospital case fatality ratios for RSV) were estimated for industrialised vs developing countries, but no European-level data were described. Age stratification and risk factor data (co-morbidities, such as cardiopulmonary and cardiovascular diseases), immunocompromised health status, and seasonality were widely reported in publications. Publications analysing RSV surveillance data in the Netherlands and the UK contained robust data on RSV-attributable mortality. Case fatality rates were estimated in literature reviews performed in French and Swedish hospitals [28, 29].

#### Staphylococcus aureus

Belgium, Denmark, Italy, the Netherlands, Norway and Spain reported using the abovementioned *S.aureus* case definition in their surveillance (Table 1). Hungary uses the official non-specific ECDC case definition of HAI, including the one for BSI [19]. Only Belgium reported that DALYs could be calculated, although only for general nosocomial infections but not for those which are pathogen-specific (Table 2). None of the surveyed countries collect disability/utility weights. All countries, except for Norway, have *S.aureus* incidence data available or data which is easily retrievable and can be stratified by risk factors. Several countries stated that they collect sequelae and hospitalisation data. Very few countries collect mortality data, while all countries collect AMR data. Additionally, some countries collect non-BSI data, such as *S.aureus* pneumonia and surgical site infection cases.

#### Literature review

The literature search yielded 185 publications, of which 101 publications remained for data extraction after full-text review (Additional files 3 and 4). Most publications came from Denmark (*n* = 14), followed by Spain (*n* = 13), Germany (*n* = 9), England (*n* = 8), France (*n* = 7), and Finland (*n* = 6). Two large European surveillance systems were identified: the EARS-Net [20], and the Central Asian and Eastern European Surveillance of Antimicrobial Resistance (CAESAR) [29]. Both collect data on blood cultures and AMR, which can be used to calculate the incidence of *S.aureus* bacteraemia. EARS-Net also allows age and gender stratification (Table 2). None of the publications reported summary BoD measures. Incidence data could be extracted from 51 publications. In most cases, data were stratified by age. Sequelae and hospitalisation data were often retrievable. Disease duration was only reported in three studies. In total, 75 studies reported on *S.aureus*-specific or all-cause mortality. Many publications included risk factor analyses related to mortality data. Almost all publications reported AMR in *S. aureus* infections.

## Discussion

This review provides a comprehensive overview of the available data sources and gaps for estimating BoD for five priority (P)VPD in ageing adults in the EU/EAA.

Our expert surveys reveal that morbidity data are generally available for the diseases of interest. Furthermore, the majority of countries collect mortality data for norovirus, PnPn and RSV, or for the associated syndromic disease, such as acute gastroenteritis. These results are corroborated by the results of the literature review, especially for *S.aureus*, ExPEC and RSV.

We found that summary BoD estimates, such as DALYs and QALYs, are rarely available for the five diseases. These summary measures are particularly valuable for understanding the health impacts from a specific (P)VPD in relation to other health conditions that affect ageing adults [30]. Additionally, some parameters are missing, such as the disability weights (DWs) needed to calculate summary BoD estimates. DWs indicate the severity of illness for a given non-fatal health outcome and are typically based on the preferences of a panel of patients or medical experts that rates the relative undesirability of hypothetical health outcomes [31, 32]. Since DWs can depend on the cultural values and distribution of health conditions that are specific to the setting in which they are elicited, their valuation should be ideally based on elicitation panels specific for the population of interest [33]. However, in practice, the analysts make use of large databases of elicited DWs covering a wide range of diseases/health conditions such as those produced for the Global Burden of Disease study [34, 35].

Another important data gap is the overall absence of completeness assessments of morbidity data. Given that population-based disease incidence and/or prevalence data are the central components of summary BoD measures, it is fundamental to assess the validity of these parameters. Notwithstanding the high quality of current EU infectious disease surveillance systems, underestimation is known to be a common issue in surveillance data. Its magnitude may vary depending on the disease, country, time period, and patient characteristics, and this variation can be due to various factors such as differences in healthcare-seeking behaviour, case management, and surveillance systems [36–38]. Estimates of underestimation may be obtained, for example, by comparing passive surveillance system data to population-based survey data. Several methods are available to adjust BoD input data and thus estimate true incidence [30].

Moreover, morbidity/incidence data were not systematically stratified by age and risk group. The lack of age-specific parameters that are needed for accurate DALY/QALY computation represents a critical data gap in the context of ageing adults. Kristensen et al. [39] studied BoD in ageing adults for four vaccine-preventable diseases and pinpointed that more precise BoD estimates could be obtained by specifying age-specific, rather than ‘age-agnostic’, indicators (i.e. overall values). It is noteworthy that stratification on age is also important for transition probabilities (e.g., the risk of developing complications following acute infection) and is expected to be larger in ageing adults, but these were not investigated in our review.

Although it is preferable to rely on country-specific data to estimate BoD, estimates for some parameters can be extrapolated from another country if no country-specific data are available. For example, case-fatality rates from one country may be applicable to other countries with similar health care systems. However, unlike case-fatality rates, incidence data usually depend greatly on the country, and therefore, country-specific incidence estimates are critical for BoD estimates.

Although we have seen that morbidity data are widely available, these data might not always be pathogen specific. For (P)VPDs, such as PnPn or norovirus, laboratory confirmation and pathogen-specific incidence data are currently scarce in EU/EEA, and, consequently, it might be challenging to estimate BoD for these pathogens. In this case, data on the incidence of a clinically-defined set of symptoms or clinical syndrome (e.g. pneumonia, gastroenteritis, etc.) need to be first attributed to the responsible pathogen(s), usually by resorting to an attributable fraction obtained from the literature, or otherwise estimated. For PnPn and norovirus, more such data are needed to accurately estimate the BoD of (P)VPDs for European countries.

For ExPEC, which is usually a hospital-detected infection, the attribution of BoD to the infection or the patient’s underlying condition poses an additional challenge for the estimation of BoD that needs to be handled. The true ExPEC infection incidence must be extrapolated from available observed cases. Indeed, infections in ageing adults are often associated with co-morbidities, and thus, are reported as secondary diagnoses in hospital care [7].

Based on studies including outpatient PnPn, the burden of PnPn is underestimated when using only hospitalised patient data [40, 41]. Therefore, the identified lack of data on outpatient pneumonia should be taken into account, and assessments of the degree of under-reporting and under-ascertainment of the incidence data are also needed. Furthermore, there is a lack of serotype-specific data, which is mainly due to methodological challenges. As long as pneumococcal vaccines do not cover all serotypes, the BoD for the vaccine-preventable part of all PnPn can only be estimated with available serotype-specific data.

In the case of ExPEC and *S.aureus*, incidence and mortality data are available but are often restricted to resistant strains. Also, summary BoD measure estimates might be underestimated because patients infected with drug-susceptible strains are not included in the calculations.

The main limitation of our study is that not all EU/EEA countries were covered by the disease expert survey. Also, the survey responses were not formally validated, even though the results were shared and discussed within the IMI2-VITAL project Consortium. In addition, although the extended literature review allowed us to capture peer-reviewed literature, only a limited and selected amount of grey literature was covered in our study. The literature reviews were restricted to English language publications, and in consequence, we may have missed some important data sources. Finally, the disease outcome trees, the duration of possible sequelae, and transition probabilities between health outcomes were not considered in our study, although these parameters would also be needed to compute summary BoD measures.

## Conclusions

Despite the fact that several methodological tools are available to overcome at least some of the data gaps identified, the results of this review have highlighted the need for more BoD studies to be conducted. Our findings may guide future studies towards specific (P)VPDs, countries and parameters for which information is currently lacking. Furthermore, all the data retrieved can be used to find references for specific BoD parameters. This information will be useful for the remainder of the IMI2-VITAL project, but may also be used to inform other BoD studies on (P)VPD. In addition, this study provides a framework that can be easily adapted to other vaccine-preventable diseases in the future.

## Supplementary Information

**Additional file 1.** Survey questionnaires.

**Additional file 2.** Search strings literature reviews.

**Additional file 3.** PRISMA flowcharts literature reviews.

**Additional file 4.** References of literature reviews.

## Data Availability

The data supporting the conclusions of this article are included within the article and its additional files.

## Abbreviations

AMR: Antimicrobial resistance
BoD: Burden of Disease
BSI: Bloodstream Infection
DALY: Disability-Adjusted Life Year
DW: Disability weight
ECDC: European Centre for Disease Prevention and Control
ExPEC: Extra-intestinal pathogenic *Escherichia coli*
HA-BSI: Hospital-Associated Bloodstream Infections
IMI: Innovative Medicine Initiative
LRTI: Lower respiratory tract infection
PnPn: Pneumococcal pneumonia
(P)VPD: (Potentially) vaccine-preventable diseases
QALY: Quality-Adjusted Life Year
QoL: Quality of Life
RSV: Respiratory syncytial virus
*S. aureus*: *Staphylococcus aureus*
URTI: Lower respiratory tract infection
VITAL: Vaccines and InfecTious Diseases in the Ageing PopuLation
YLD: Years Lived with Disability
YLL: Years of Life Lost
