# Supplementary material for: European data sources for computing burden of (potential) vaccine-preventable diseases in ageing adults
Source: BMC Infect Dis. 2021 Apr 13;21:345. doi: 10.1186/s12879-021-06017-7 (PMC8042717; doi:10.1186/s12879-021-06017-7)
Supplement: Supplementary file 1 — Additional file 1. Survey questionnaires. [file 12879_2021_6017_MOESM1_ESM.docx]

**Additional file 1: Survey questionnaires**

**Extra-intestinal pathogenic Escherichia coli :**

### Norovirus:

### Pneumococcal Pneumonia:

### Respiratory syncytial virus:

### Staphylococcus aureus:
