# Supplementary material for: European data sources for computing burden of (potential) vaccine-preventable diseases in ageing adults
Source: BMC Infect Dis. 2021 Apr 13;21:345. doi: 10.1186/s12879-021-06017-7 (PMC8042717; doi:10.1186/s12879-021-06017-7)
Supplement: Supplementary file 2 — Additional file 2. Search strings literature reviews. [file 12879_2021_6017_MOESM2_ESM.docx]

**Additional file 2: Search strings literature reviews**

**Extra-intestinal pathogenic Escherichia coli:**

Final search string used for PubMed literature review on ExPEC, with number of results.

| "Health related quality of life" [tiab] OR "Hrqol"[tiab] OR "Quality of life" [Mesh] OR "burden of disease"[tiab] OR "BoD" [tiab] OR "Global disease burden" [Mesh] OR "Global disease burden" [tiab] OR "disease burden" [tiab] OR "BeCoDe" [tiab] OR "Burden of communicable diseases" [tiab] OR disability adjusted life year*[tiab]OR "DALY"[tiab] OR "years of life lost" [tiab] OR "YLL"[tiab] OR "YLD" [tiab])OR Years of life lost due to disabilit*[tiab] OR disability weight*[tiab] OR Summary Health Measure*[tiab] OR "SMPH" [tiab])OR "SMPHs" [tiab] OR “Quality of Life”[Mesh] OR “Patient Satisfaction”[Mesh] OR “Pain Measurement”[Mesh] OR “Activities of Daily Living”[Mesh] OR “Patient Compliance”[Mesh] OR “quality of life”[Title] OR “QoL”[Title] OR “hrqol”[Title] OR “hqol”[Title] OR “hrql”[Title] OR patient report*[Title] OR “self report”[Title] OR “health utility”[Title] OR “health utilities”[Title] OR “EuroQol”[Title] OR “EQ5D”[Title] OR “EQ 5D”[Title] OR “quality of well being”[Title] OR “QALY” OR “quality adjusted life year”[Title] OR “quality adjusted life years”[Title] OR “activities of daily living”[Title] OR “patient satisfaction”[Title] OR burden[Title] OR “health status”[Title]) OR (((((incidence[MeSH Terms]) OR incidence[Title/Abstract]) OR epidemiology[Title/Abstract]) OR epidemiology[MeSH Terms]) OR surveillance[MeSH Terms]) OR surveillance[Title/Abstract] AND "Aged" [Mesh] OR "Middle Aged" [Mesh] OR "Aged" [tiab] OR "Middle aged" [tiab] OR "elderly" [tiab] OR frail* [tiab] OR “frail elderly” [Mesh] AND (Extraintestinal Pathogenic Escherichia coli [Mesh] OR Extraintestinal Pathogenic Escherichia coli [tiab] OR Extraintestinal Pathogenic E. coli [tiab] OR Escherichia coli Infections [mesh] OR Escherichia coli Infections [tiab] OR Gram-Negative Bacterial Infections [mesh]) OR Gram-Negative Bacterial Infections [tiab] OR Escherichia coli [tiab] OR E. coli [tiab])) AND (bacteremia [mesh] OR bacteremia [tiab] OR sepsis [mesh] OR sepsis [tiab] OR blood infection [tiab] OR bloodstream infection [tiab]) | 1372 results |
| --- | --- |

### Norovirus:

Final search string used for PubMed literature review on norovirus, with number of results.

| (((("Health related quality of life" [tiab] OR "Hrqol"[tiab] OR "Quality of life" [Mesh] OR "burden of disease"[tiab] OR "BoD" [tiab] OR "Global disease burden" [Mesh] OR "Global disease burden" [tiab] OR "disease burden" [tiab] OR "BeCoDe" [tiab] OR "Burden of communicable diseases" [tiab] OR disability adjusted life year*[tiab]OR "DALY"[tiab] OR "years of life lost" [tiab] OR "YLL"[tiab] OR "YLD" [tiab])OR Years of life lost due to disabilit*[tiab] OR disability weight*[tiab] OR Summary Health Measure*[tiab] OR "SMPH" [tiab])OR "SMPHs" [tiab])) OR (“Quality of Life”[Mesh] OR “Patient Satisfaction”[Mesh] OR “Pain Measurement”[Mesh] OR “Activities of Daily Living”[Mesh] OR “Patient Compliance”[Mesh] OR “quality of life”[Title] OR “QoL”[Title] OR “hrqol”[Title] OR “hqol”[Title] OR “hrql”[Title] OR patient report*[Title] OR “self report”[Title] OR “health utility”[Title] OR “health utilities”[Title] OR “EuroQol”[Title] OR “EQ5D”[Title] OR “EQ 5D”[Title] OR “quality of well being”[Title] OR “QALY” OR “quality adjusted life year”[Title] OR “quality adjusted life years”[Title] OR “activities of daily living”[Title] OR “patient satisfaction”[Title] OR burden[Title] OR “health status”[Title]))) OR (incidence [tiab] AND epidemiology [tiab]AND surveillance [tiab])) AND "Aged" [Mesh] OR "Middle Aged" [Mesh]) OR "Aged" [tiab] OR "Middle aged" [tiab] OR "elderly" [tiab] OR frail* [tiab] OR “frail elderly” [Mesh]  AND Norovirus”[Mesh] OR “Norovirus” [ti] OR “Norwalk virus” [tiab] OR “Norwalk-like virus” [tiab] OR norovirus* [tiab] OR “Caliciviridae Infections/epidemiology*” [Mesh] or “Gastroenteritis/epidemiology*”[Mesh] OR gastroenteritis [tiab] OR viral gastroenteritis [tiab] Filters: published in the last 10 years | 364 results |
| --- | --- |

### Pneumococcal Pneumonia:

Final search string used for PubMed literature review on pneumococcal pneumonia, with number of results.

| (((inciden*[tiab] OR epidemiolog*[tiab] OR surveillance[tiab] OR rate[tiab] OR mortality[tiab] OR (case[tiab] AND fatality[tiab]) OR morbidity[tiab] OR “attributable fraction”[tiab]) AND (“pneumococcal infections”[MeSH] OR “strep pneumoniae” [MeSH] OR “Streptococcus pneumoniae”[all fields] OR “S pneumoniae”[all fields] OR “Streptococcus pneumonie”[all fields] OR "pneumococcus"[all fields] OR "pneumococcal"[all fields] OR "pneumococci"[all fields])) OR"Health related quality of life"[tiab] OR "Hrqol"[tiab] OR "Quality of life"[Mesh] OR "burden of disease"[tiab] OR "BoD"[tiab] OR "Global Burden of Disease"[Mesh] OR "Global disease burden"[tiab] OR "disease burden"[tiab] OR "BeCoDe"[tiab] OR "Burden of communicable diseases"[tiab] OR disability adjusted life year*[tiab] OR "DALY"[tiab] OR "years of life lost"[tiab] OR "YLL"[tiab] OR "YLD"[tiab] OR (Years of life lost due to disabilit*[tiab]) OR (disability weight*[tiab]) OR (Summary Health Measure*[tiab]) OR "SMPH"[tiab] OR "SMPHs"[tiab] OR "Quality of Life"[Mesh] OR "Patient Satisfaction"[Mesh] OR "Pain Measurement"[Mesh] OR "Activities of Daily Living"[Mesh] OR "Patient Compliance"[Mesh] OR "quality of life"[Title] OR "QoL"[Title] OR "hrqol"[Title] OR "hqol"[Title] OR "hrql"[Title] OR patient report*[Title] OR "self report"[Title] OR "health utility"[Title] OR "health utilities"[Title] OR "EuroQol"[Title] OR "EQ5D"[Title] OR "EQ 5D"[Title] OR "quality of well being"[Title] OR "QALY"[Title] OR "quality adjusted life year"[Title] OR "quality adjusted life years"[Title] OR "activities of daily living"[Title] OR "patient satisfaction"[Title] OR burden[Title] OR "health status"[Title]) AND ("pneumonia, pneumococcal"[Mesh] OR Pneumonia[MeSH] OR "community acquired pneumonia"[tiab]) AND ("Aged"[Mesh] OR "Middle Aged"[Mesh] OR "Aged"[tiab] OR "Middle aged"[tiab] OR "elderly"[tiab] OR frail*[tiab] OR "frail elderly"[Mesh]) AND ("2012"[Date - Publication] : "3000"[Date - Publication]) | 778 results |
| --- | --- |

### Respiratory syncytial virus:

Final search string used for PubMed literature review on RSV, with number of results.

| (((((((("Health related quality of life" [tiab] OR "Hrqol"[tiab] OR "Quality of life" [Mesh] OR "burden of disease"[tiab] OR "BoD" [tiab] OR "Global disease burden" [Mesh] OR "Global disease burden" [tiab] OR "disease burden" [tiab] OR "BeCoDe" [tiab] OR "Burden of communicable diseases" [tiab] OR disability adjusted life year*[tiab]OR "DALY"[tiab] OR "years of life lost" [tiab] OR "YLL"[tiab] OR "YLD" [tiab])OR Years of life lost due to disabilit*[tiab] OR disability weight*[tiab] OR Summary Health Measure*[tiab] OR "SMPH" [tiab])OR "SMPHs" [tiab]))) OR ((“Quality of Life”[Mesh] OR “Patient Satisfaction”[Mesh] OR “Pain Measurement”[Mesh] OR “Activities of Daily Living”[Mesh] OR “Patient Compliance”[Mesh] OR “quality of life”[Title] OR “QoL”[Title] OR “hrqol”[Title] OR “hqol”[Title] OR “hrql”[Title] OR patient report*[Title] OR “self report”[Title] OR “health utility”[Title] OR “health utilities”[Title] OR “EuroQol”[Title] OR “EQ5D”[Title] OR “EQ 5D”[Title] OR “quality of well being”[Title] OR “QALY” OR “quality adjusted life year”[Title] OR “quality adjusted life years”[Title] OR “activities of daily living”[Title] OR “patient satisfaction”[Title] OR burden[Title] OR “health status”[Title])))) OR ((inciden*[tiab] OR prevalen*[tiab] OR epidemiolog*[tiab] OR surveillance[tiab] OR rate[tiab] OR progression[tiab] OR outcomes[tiab] OR mortality[tiab] OR (case[tiab] AND fatality[tiab]) OR morbidity[tiab] OR complication[tiab] OR “attributable fraction”[tiab])))) AND (("Aged" [Mesh] OR "Middle Aged" [Mesh]) OR "Aged" [tiab] OR "Middle aged" [tiab] OR "elderly" [tiab] OR frail* [tiab] OR “frail elderly” [Mesh])))) AND ((“Respiratory Syncytial Virus, Human”[Mesh] OR “Respiratory Syncytial Virus Infections”[Mesh] OR “Respiratory Syncytial Virus”[Title] OR “RSV”[Title] OR (“Respiratory Tract Infections”[Mesh] AND (syncytial[Text Word] OR RSV [Text Word])))) | 1123 results |
| --- | --- |

### Staphylococcus aureus:

Final search string used for PubMed literature review on *S. aureus* bacteraemia, with number of results.

| (((((((((("Health related quality of life"[tiab] OR "Hrqol"[tiab] OR "Quality of life"[Mesh] OR "burden of disease"[tiab] OR "BoD"[tiab] OR "Global disease burden"[Mesh] OR "Global disease burden"[tiab] OR "disease burden"[tiab] OR "BeCoDe"[tiab] OR "Burden of communicable diseases"[tiab] OR disability adjusted life year*[tiab] OR "DALY"[tiab] OR "years of life lost"[tiab] OR "YLL"[tiab] OR "YLD"[tiab]) OR Years of life lost due to disabilit*[tiab] OR disability weight*[tiab] OR Summary Health Measure*[tiab] OR "SMPH"[tiab]) OR "SMPHs"[tiab]))) OR (("Quality of Life"[Mesh] OR "Patient Satisfaction"[Mesh] OR "Pain Measurement"[Mesh] OR "Activities of Daily Living"[Mesh] OR "Patient Compliance"[Mesh] OR "quality of life"[Title] OR "QoL"[Title] OR "hrqol"[Title] OR "hqol"[Title] OR "hrql"[Title] OR patient report*[Title] OR "self report"[Title] OR "health utility"[Title] OR "health utilities"[Title] OR "EuroQol"[Title] OR "EQ5D"[Title] OR "EQ 5D"[Title] OR "quality of well being"[Title] OR "QALY" OR "quality adjusted life year"[Title] OR "quality adjusted life years"[Title] OR "activities of daily living"[Title] OR "patient satisfaction"[Title] OR burden[Title] OR "health status"[Title])))) OR ((inciden*[tiab] OR prevalen*[tiab] OR epidemiolog*[tiab] OR surveillance[tiab] OR rate[tiab] OR progression[tiab] OR outcomes[tiab] OR mortality[tiab] OR (case[tiab] AND fatality[tiab]) OR morbidity[tiab] OR complication[tiab] OR "attributable fraction"[tiab]))) AND ((((("Staphylococcus aureus"[Mesh] OR Staphylococcus aureus[tiab] OR S. Aureus*[tiab])))) AND (((bacteremia[mesh] OR bacteremia[tiab] OR sepsis[mesh] OR sepsis[tiab] OR blood infection[tiab] OR bloodstream infection[tiab]))) AND (("Aged"[Mesh] OR "Middle Aged"[Mesh]) OR "Aged"[tiab] OR "Middle aged"[tiab] OR "elderly"[tiab] OR frail*[tiab] OR "frail elderly"[Mesh])))) AND ("last 10 years"[PDat]) | 1175 results |
| --- | --- |
