# Supplementary material for: European data sources for computing burden of (potential) vaccine-preventable diseases in ageing adults
Source: BMC Infect Dis. 2021 Apr 13;21:345. doi: 10.1186/s12879-021-06017-7 (PMC8042717; doi:10.1186/s12879-021-06017-7)

**Additional file 3: PRISMA flowcharts literature reviews**


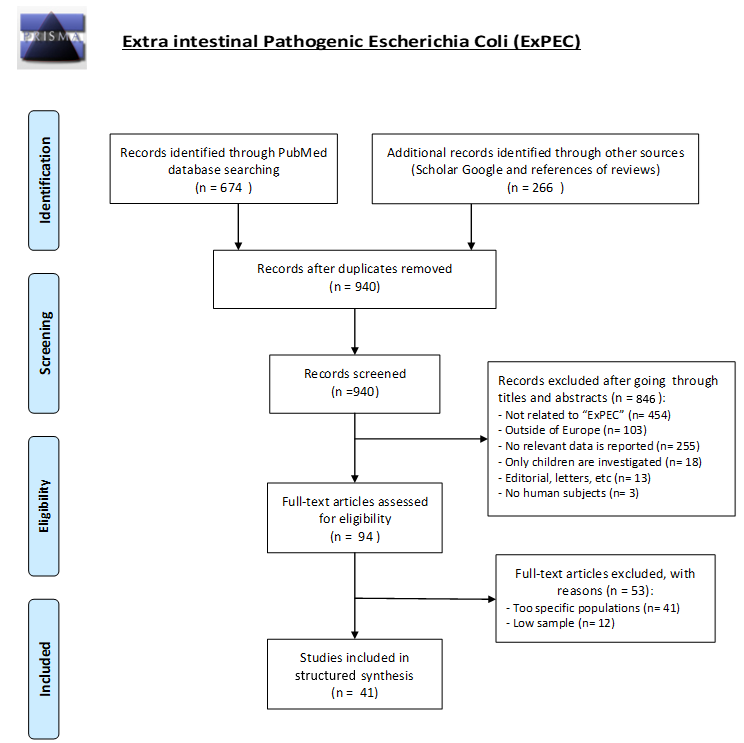

**Pneumococcal Pneumonia**


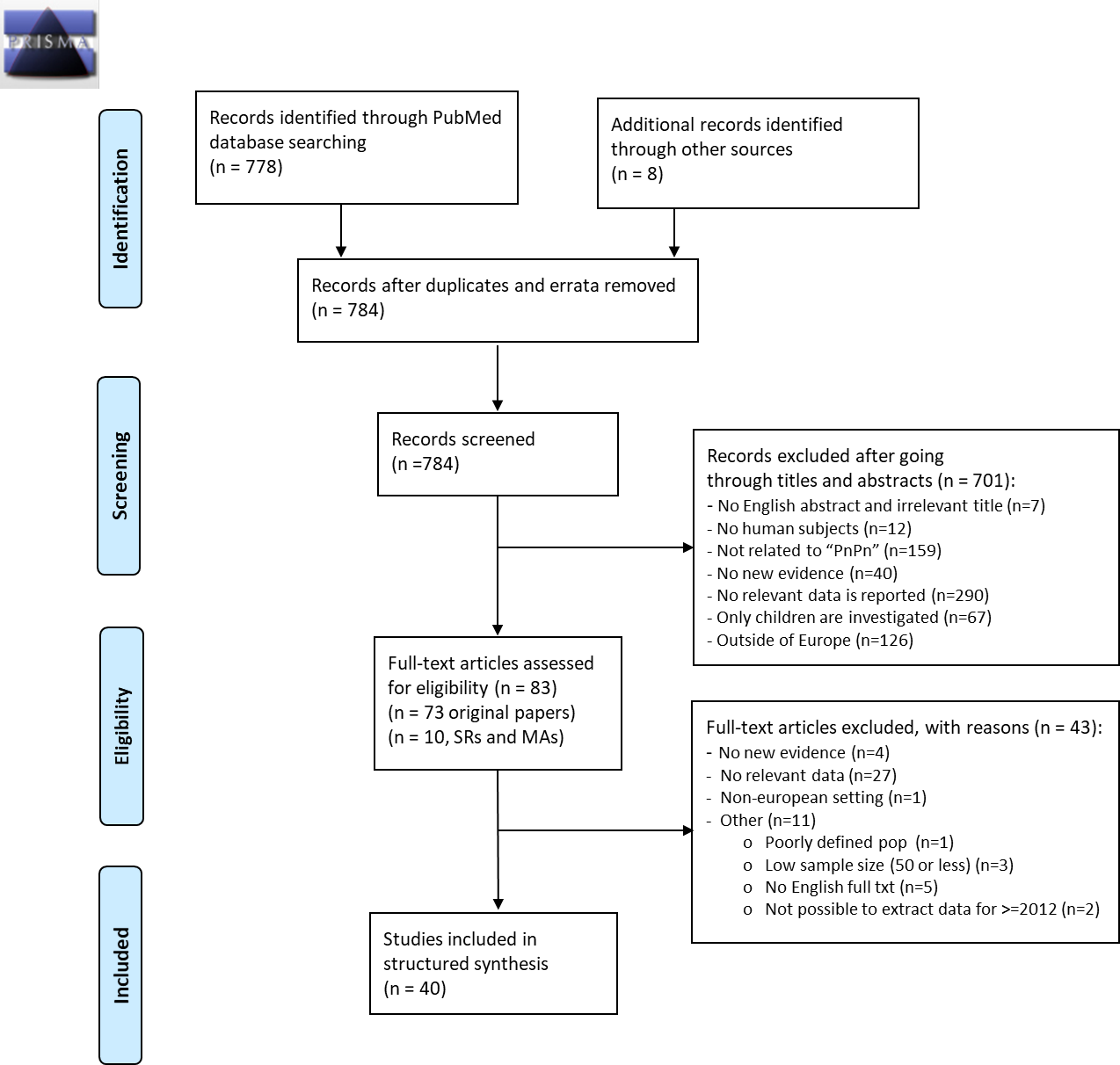


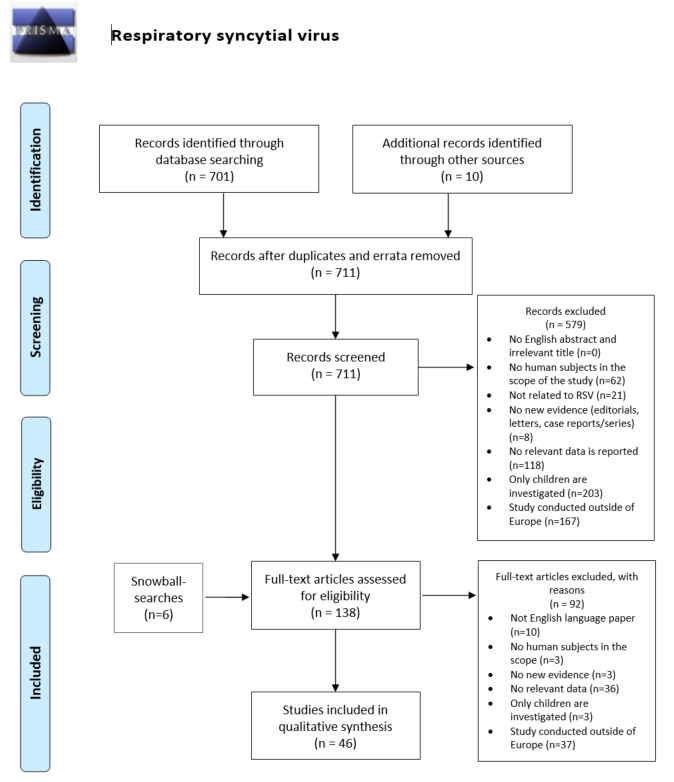


**Staphylococcus aureus**


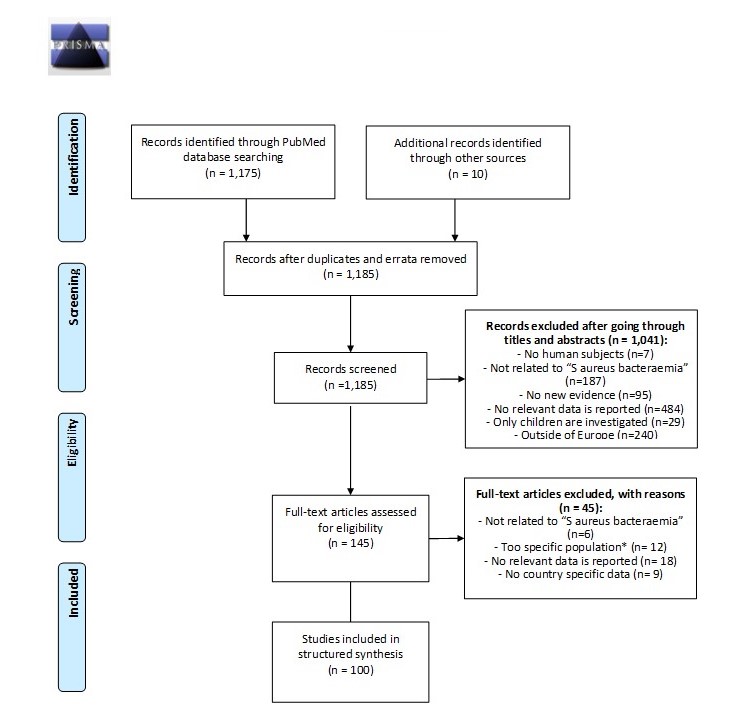

Supplement: Supplementary file 3 — Additional file 3. PRISMA flowcharts literature reviews. [file 12879_2021_6017_MOESM3_ESM.docx]
