# Supplementary material for: European data sources for computing burden of (potential) vaccine-preventable diseases in ageing adults
Source: BMC Infect Dis. 2021 Apr 13;21:345. doi: 10.1186/s12879-021-06017-7 (PMC8042717; doi:10.1186/s12879-021-06017-7)
Supplement: Supplementary file 4 — Additional file 4. References of literature reviews. [file 12879_2021_6017_MOESM4_ESM.docx]

**Additional file 4: References of literature reviews**

**Extra-intestinal pathogenic Escherichia coli:**

1. Blandy O, Honeyford K, Gharbi M, Thomas A, Ramzan F, Ellington MJ, et al. Factors that impact on the burden of Escherichia coli bacteraemia: multivariable regression analysis of 2011-2015 data from West London. J Hosp Infect. 2019;101(2):120-8.

2. Lishman H, Costelloe C, Hopkins S, Johnson AP, Hope R, Guy R, et al. Exploring the relationship between primary care antibiotic prescribing for urinary tract infections, Escherichia coli bacteraemia incidence and antimicrobial resistance: an ecological study. Int J Antimicrob Agents. 2018;52(6):790-8.

3. Bhattacharya A, Nsonwu O, Johnson AP, Hope R. Estimating the incidence and 30-day all-cause mortality rate of Escherichia coli bacteraemia in England by 2020/21. J Hosp Infect. 2018;98(3):228-31.

4. Martin Jaramago J, Armero Ibanez R, Camarena Minana JJ, Morales Suarez-Varela M. Resistance profiles and risk factors of resistant microorganisms in bacteraemia of abdominal origin. Rev Esp Anestesiol Reanim. 2017;64(9):490-8.

5. Weisser M, Theilacker C, Tschudin Sutter S, Babikir R, Bertz H, Gotting T, et al. Secular trends of bloodstream infections during neutropenia in 15 181 haematopoietic stem cell transplants: 13-year results from a European multicentre surveillance study (ONKO-KISS). Clin Microbiol Infect. 2017;23(11):854-9.

6. Buetti N, Atkinson A, Marschall J, Kronenberg A. Incidence of bloodstream infections: a nationwide surveillance of acute care hospitals in Switzerland 2008-2014. BMJ Open. 2017;7(3):e013665.

7. Mehl A, Asvold BO, Lydersen S, Paulsen J, Solligard E, Damas JK, et al. Burden of bloodstream infection in an area of Mid-Norway 2002-2013: a prospective population-based observational study. BMC Infect Dis. 2017;17(1):205.

8. Sogaard KK, Farkas DK, Sogaard M, Schonheyder HC, Thomsen RW, Sorensen HT. Gram-negative bacteremia as a clinical marker of occult malignancy. J Infect. 2017;74(2):153-62.

9. Holmbom M, Giske CG, Fredrikson M, Ostholm Balkhed A, Claesson C, Nilsson LE, et al. 14-Year Survey in a Swedish County Reveals a Pronounced Increase in Bloodstream Infections (BSI). Comorbidity - An Independent Risk Factor for Both BSI and Mortality. PLoS One. 2016;11(11):e0166527.

10. Bou-Antoun S, Davies J, Guy R, Johnson AP, Sheridan EA, Hope RJ. Descriptive epidemiology of Escherichia coli bacteraemia in England, April 2012 to March 2014. Euro Surveill. 2016;21(35).

11. Stewardson AJ, Allignol A, Beyersmann J, Graves N, Schumacher M, Meyer R, et al. The health and economic burden of bloodstream infections caused by antimicrobial-susceptible and non-susceptible Enterobacteriaceae and Staphylococcus aureus in European hospitals, 2010 and 2011: a multicentre retrospective cohort study. Euro Surveill. 2016;21(33).

12. Kontula KS, Skogberg K, Ollgren J, Jarvinen A, Lyytikainen O. Early deaths in bloodstream infections: a population-based case series. Infect Dis (Lond). 2016;48(5):379-85.

13. Martelius T, Jalava J, Karki T, Mottonen T, Ollgren J, Lyytikainen O. Nosocomial bloodstream infections caused by Escherichia coli and Klebsiella pneumoniae resistant to third-generation cephalosporins, Finland, 1999-2013: Trends, patient characteristics and mortality. Infect Dis (Lond). 2016;48(3):229-34.

14. Poolman JT, Anderson AS. Escherichia coli and Staphylococcus aureus: leading bacterial pathogens of healthcare associated infections and bacteremia in older-age populations. Expert Rev Vaccines. 2018;17(7):607-18.

15. van der Mee-Marquet NL, Blanc DS, Gbaguidi-Haore H, Dos Santos Borges S, Viboud Q, Bertrand X, et al. Marked increase in incidence for bloodstream infections due to Escherichia coli, a side effect of previous antibiotic therapy in the elderly. Front Microbiol. 2015;6:646.

16. Abernethy JK, Johnson AP, Guy R, Hinton N, Sheridan EA, Hope RJ. Thirty day all-cause mortality in patients with Escherichia coli bacteraemia in England. Clin Microbiol Infect. 2015;21(3):251.e1-8.

17. Gubbels S, Nielsen J, Voldstedlund M, Kristensen B, Schonheyder HC, Vandenbroucke-Grauls CM, et al. Utilization of blood cultures in Danish hospitals: a population-based descriptive analysis. Clin Microbiol Infect. 2015;21(4):344.e13-21.

18. Hoenigl M, Wagner J, Raggam RB, Prueller F, Prattes J, Eigl S, et al. Characteristics of hospital-acquired and community-onset blood stream infections, South-East Austria. PLoS One. 2014;9(8):e104702.

19. Leistner R, Gurntke S, Sakellariou C, Denkel LA, Bloch A, Gastmeier P, et al. Bloodstream infection due to extended-spectrum beta-lactamase (ESBL)-positive K. pneumoniae and E. coli: an analysis of the disease burden in a large cohort. Infection. 2014;42(6):991-7.

20. Shoai Tehrani M, Hajage D, Fihman V, Tankovic J, Cau S, Day N, et al. Gram-negative bacteremia: which empirical antibiotic therapy? Med Mal Infect. 2014;44(4):159-66.

21. Nielsen SL, Pedersen C, Jensen TG, Gradel KO, Kolmos HJ, Lassen AT. Decreasing incidence rates of bacteremia: a 9-year population-based study. J Infect. 2014;69(1):51-9.

22. Ortega M, Marco F, Soriano A, Almela M, Martinez JA, Pitart C, et al. Epidemiology and prognostic determinants of bacteraemic catheter-acquired urinary tract infection in a single institution from 1991 to 2010. J Infect. 2013;67(4):282-7.

23. Skjot-Rasmussen L, Ejrnaes K, Lundgren B, Hammerum AM, Frimodt-Moller N. Virulence factors and phylogenetic grouping of Escherichia coli isolates from patients with bacteraemia of urinary tract origin relate to sex and hospital- vs. community-acquired origin. Int J Med Microbiol. 2012;302(3):129-34.

24. Skogberg K, Lyytikainen O, Ollgren J, Nuorti JP, Ruutu P. Population-based burden of bloodstream infections in Finland. Clin Microbiol Infect. 2012;18(6):E170-6.

25. Schlackow I, Stoesser N, Walker AS, Crook DW, Peto TE, Wyllie DH. Increasing incidence of Escherichia coli bacteraemia is driven by an increase in antibiotic-resistant isolates: electronic database study in Oxfordshire 1999-2011. J Antimicrob Chemother. 2012;67(6):1514-24.

26. Underwood J, Klein JL, Newsholme W. Escherichia coli bacteraemia: how preventable is it? J Hosp Infect. 2011;79(4):364-5.

27. Luzzaro F, Ortisi G, Larosa M, Drago M, Brigante G, Gesu G. Prevalence and epidemiology of microbial pathogens causing bloodstream infections: results of the OASIS multicenter study. Diagn Microbiol Infect Dis. 2011;69(4):363-9.

28. Cuevas O, Oteo J, Lazaro E, Aracil B, de Abajo F, Garcia-Cobos S, et al. Significant ecological impact on the progression of fluoroquinolone resistance in Escherichia coli with increased community use of moxifloxacin, levofloxacin and amoxicillin/clavulanic acid. J Antimicrob Chemother. 2011;66(3):664-9.

29. Lambert ML, Suetens C, Savey A, Palomar M, Hiesmayr M, Morales I, et al. Clinical outcomes of health-care-associated infections and antimicrobial resistance in patients admitted to European intensive-care units: a cohort study. Lancet Infect Dis. 2011;11(1):30-8.

30. de Kraker ME, Wolkewitz M, Davey PG, Koller W, Berger J, Nagler J, et al. Burden of antimicrobial resistance in European hospitals: excess mortality and length of hospital stay associated with bloodstream infections due to Escherichia coli resistant to third-generation cephalosporins. J Antimicrob Chemother. 2011;66(2):398-407.

31. Wilson J, Elgohari S, Livermore DM, Cookson B, Johnson A, Lamagni T, et al. Trends among pathogens reported as causing bacteraemia in England, 2004-2008. Clin Microbiol Infect. 2011;17(3):451-8.

32. Rodriguez-Bano J, Lopez-Prieto MD, Portillo MM, Retamar P, Natera C, Nuno E, et al. Epidemiology and clinical features of community-acquired, healthcare-associated and nosocomial bloodstream infections in tertiary-care and community hospitals. Clin Microbiol Infect. 2010;16(9):1408-13.

33. Buetti N, Marschall J, Atkinson A, Kronenberg A. National Bloodstream Infection Surveillance in Switzerland 2008-2014: Different Patterns and Trends for University and Community Hospitals. Infect Control Hosp Epidemiol. 2016;37(9):1060-7.

34. Alfandari S, Cabaret P, Nguyen S, Descamps D, Vachee A, Cattoen C, et al. Evaluating the management of 493 patients presenting with bacteremia in 23 northern French hospitals. Med Mal Infect. 2016;46(4):194-9.

35. Nielsen SL. The incidence and prognosis of patients with bacteremia. Dan Med J. 2015;62(7).

36. Bukh AS, Schonheyder HC, Emmersen JM, Sogaard M, Bastholm S, Roslev P. Escherichia coli phylogenetic groups are associated with site of infection and level of antibiotic resistance in community-acquired bacteraemia: a 10 year population-based study in Denmark. J Antimicrob Chemother. 2009;64(1):163-8.

37. Lillie PJ, Johnson G, Ivan M, Barlow GD, Moss PJ. Escherichia coli bloodstream infection outcomes and preventability: a six-month prospective observational study. J Hosp Infect. 2019;103(2):128-33.

38. Diekema DJ, Hsueh PR, Mendes RE, Pfaller MA, Rolston KV, Sader HS, et al. The Microbiology of Bloodstream Infection: 20-Year Trends from the SENTRY Antimicrobial Surveillance Program. Antimicrob Agents Chemother. 2019;63(7).

39. Tandogdu Z, Bartoletti R, Cai T, Cek M, Grabe M, Kulchavenya E, et al. Antimicrobial resistance in urosepsis: outcomes from the multinational, multicenter global prevalence of infections in urology (GPIU) study 2003-2013. World J Urol. 2016;34(8):1193-200.

40. Cassini A, Hogberg LD, Plachouras D, Quattrocchi A, Hoxha A, Simonsen GS, et al. Attributable deaths and disability-adjusted life-years caused by infections with antibiotic-resistant bacteria in the EU and the European Economic Area in 2015: a population-level modelling analysis. Lancet Infect Dis. 2019;19(1):56-66.

**Norovirus:**

41. Arena C, Amoros JP, Vaillant V, Ambert-Balay K, Chikhi-Brachet R, Jourdan-Da Silva N, et al. Acute diarrhea in adults consulting a general practitioner in France during winter: incidence, clinical characteristics, management and risk factors. BMC infectious diseases. 2014;14:574.

42. Arias C, Sala M, Dominguez A, Torner N, Ruiz L, Martinez A, et al. Epidemiological and clinical features of norovirus gastroenteritis in outbreaks: a population-based study. Clinical Microbiology and Infection. 2010;16(1):39-44.

43. Bernard H, Hohne M, Niendorf S, Altmann D, Stark K. Epidemiology of norovirus gastroenteritis in Germany 2001-2009: eight seasons of routine surveillance. Epidemiology and infection. 2014;142(1):63-74.

44. Doorduyn Y, Van Pelt W, Havelaar AH. The burden of infectious intestinal disease (IID) in the community: a survey of self-reported IID in The Netherlands. Epidemiology and infection. 2012;140(7):1185-92.

45. Friesema IHM, De Boer RF, Duizer E, Kortbeek LM, Notermans DW, Smeulders A, et al. Aetiology of acute gastroenteritis in adults requiring hospitalization in The Netherlands. Epidemiology and infection. 2012;140(10):1780-6.

46. Gustavsson L, Andersson L-M, Lindh M, Westin J. Excess mortality following community-onset norovirus enteritis in the elderly. Journal of Hospital Infection. 2011;79(1):27-31.

47. Harris JP, Iturriza-Gomara M, O’Brien SJ. Estimating Disability-Adjusted Life Years (DALYs) in Community Cases of Norovirus in England. Viruses. 2019;11(2):184.

48. Harris JP, Iturriza-Gomara M, O'Brien SJ. Re-assessing the total burden of norovirus circulating in the United Kingdom population. Vaccine. 2017;35(6):853-5.

49. Hauri AM, Uphoff H, Gawrich S. [The burden of acute gastrointestinal illness in Hesse--a telephone survey]2011 2011-Feb-. 78-84 p.

50. Haustein T, Harris JP, Pebody R, Lopman BA. Hospital admissions due to norovirus in adult and elderly patients. Clinical Infectious Diseases. 2009;49(12):1890-2.

51. Havelaar AH, Haagsma JA, Mangen M-JJ, Kemmeren JM, Verhoef LPB, Vijgen SMC, et al. Disease burden of foodborne pathogens in the Netherlands, 2009. International journal of food microbiology. 2012;156(3):231-8.

52. Kowalzik F, Binder H, Zoller D, Riera-Montes M, Clemens R, Verstraeten T, et al. Norovirus Gastroenteritis among Hospitalized Patients, Germany, 2007-2012. Emerging infectious diseases. 2018;24(11):2021-8.

53. Phillips G, Tam CC, Conti S, Rodrigues LC, Brown D, Iturriza-Gomara M, et al. Community incidence of norovirus-associated infectious intestinal disease in England: improved estimates using viral load for norovirus diagnosis2010 2010-May-01. 1014-22 p.

54. Pijnacker R, Mangen MJ, van den Bunt G, Franz E, van Pelt W, Mughini-Gras L. Incidence and economic burden of community-acquired gastroenteritis in the Netherlands: Does having children in the household make a difference? PloS one. 2019;14(5):e0217347.

55. Scavia G, Baldinelli F, Busani L, Caprioli A. The burden of self-reported acute gastrointestinal illness in Italy: a retrospective survey, 2008-2009. Epidemiology and infection. 2012;140(7):1193-206.

56. Schmutz C, Bless PJ, Mäusezahl D, Jost M, Mäusezahl-Feuz M, Swiss Sentinel Surveillance Network. Acute gastroenteritis in primary care: a longitudinal study in the Swiss Sentinel Surveillance Network, Sentinella. Infection. 2017;45(6):811-24.

57. Van Cauteren D, De Valk H, Vaux S, Le Strat Y, Vaillant V. Burden of acute gastroenteritis and healthcare-seeking behaviour in France: a population-based study. Epidemiology and infection. 2012;140(4):697-705.

58. Van Cauteren D, Turbelin C, Fonteneau L, Hanslik T, De Valk H, Blanchon T. Physician practices in requesting stool samples for patients with acute gastroenteritis, France, August 2013-July 2014. Epidemiology and infection. 2015;143(12):2532-8.

59. van Lier A, McDonald SA, Bouwknegt M, EPI group, Kretzschmar ME, Havelaar AH, et al. Disease Burden of 32 Infectious Diseases in the Netherlands, 2007-2011. PloS one. 2016;11(4):e0153106.

60. Verhoef L, Koopmans M, VAN Pelt W, Duizer E, Haagsma J, Werber D, et al. The estimated disease burden of norovirus in The Netherlands. Epidemiology and infection. 2013;141(3):496-506.

61. Verstraeten T, Cattaert T, Harris J, Lopman B, Tam CC, Ferreira G. Estimating the Burden of Medically Attended Norovirus Gastroenteritis: Modeling Linked Primary Care and Hospitalization Datasets. The Journal of infectious diseases. 2017;216(8):957-65.

62. Viviani L, van der Es M, Irvine L, Tam CC, Rodrigues LC, Jackson KA, et al. Estimating the Incidence of Acute Infectious Intestinal Disease in the Community in the UK: A Retrospective Telephone Survey. PloS one. 2016;11(1):e0146171.

63. Walter F, Ott JJ, Claus H, Krause G. Sex- and age patterns in incidence of infectious diseases in Germany: analyses of surveillance records over a 13-year period (2001-2013). Epidemiology and infection. 2018;146(3):372-8.

64. Werber D, Hille K, Frank C, Dehnert M, Altmann D, Müller-Nordhorn J, et al. Years of potential life lost for six major enteric pathogens, Germany, 2004–2008. Epidemiology & Infection. 2013;141(5):961-8.

**Pneumococcal pneumonia:**

65. Amodio E, Costantino C, Boccalini S, Tramuto F, Maida CM, Vitale F. Estimating the burden of hospitalization for pneumococcal pneumonia in a general population aged 50 years or older and implications for vaccination strategies. Human vaccines & immunotherapeutics. 2014;10(5):1337-42.

66. Andrade LF, Saba G, Ricard JD, Messika J, Gaillat J, Bonnin P, et al. Health related quality of life in patients with community-acquired pneumococcal pneumonia in France. Health and quality of life outcomes. 2018;16(1):28.

67. Baldo V, Cocchio S, Baldovin T, Buja A, Furlan P, Bertoncello C, et al. A population-based study on the impact of hospitalization for pneumonia in different age groups. BMC Infect Dis. 2014;14:485.

68. Baldo V, Cocchio S, Gallo T, Furlan P, Romor P, Bertoncello C, et al. Pneumococcal Conjugated Vaccine Reduces the High Mortality for Community-Acquired Pneumonia in the Elderly: an Italian Regional Experience. PLoS One. 2016;11(11):e0166637.

69. Baldo V, Cocchio S, Gallo T, Furlan P, Clagnan E, Del Zotto S, et al. Impact of pneumococcal conjugate vaccination: a retrospective study of hospitalization for pneumonia in North-East Italy. Journal of preventive medicine and hygiene. 2016;57(2):E61-8.

70. Bechini A, Taddei C, Barchielli A, Levi M, Tiscione E, Santini MG, et al. A retrospective analysis of hospital discharge records for S. pneumoniae diseases in the elderly population of Florence, Italy, 2010-2012. Human vaccines & immunotherapeutics. 2015;11(1):156-65.

71. Cilloniz C, Ewig S, Gabarrus A, Ferrer M, Puig de la Bella Casa J, Mensa J, et al. Seasonality of pathogens causing community-acquired pneumonia. Respirology (Carlton, Vic). 2017;22(4):778-85.

72. Cilloniz C, Liapikou A, Martin-Loeches I, Garcia-Vidal C, Gabarrus A, Ceccato A, et al. Twenty-year trend in mortality among hospitalized patients with pneumococcal community-acquired pneumonia. PLoS One. 2018;13(7):e0200504.

73. de Miguel-Diez J, Jimenez-Garcia R, Hernandez-Barrera V, Jimenez-Trujillo I, de Miguel-Yanes JM, Mendez-Bailon M, et al. Trends in hospitalizations for community-acquired pneumonia in Spain: 2004 to 2013. European journal of internal medicine. 2017;40:64-71.

74. de Miguel-Diez J, Lopez-de-Andres A, Hernandez-Barrera V, Jimenez-Trujillo I, Mendez-Bailon M, de Miguel-Yanes JM, et al. Impact of COPD on outcomes in hospitalized patients with community-acquired pneumonia: Analysis of the Spanish national hospital discharge database (2004-2013). European journal of internal medicine. 2017;43:69-76.

75. Flamaing J, De Backer W, Van Laethem Y, Heijmans S, Mignon A. Pneumococcal lower respiratory tract infections in adults: an observational case-control study in primary care in Belgium. BMC family practice. 2015;16:66.

76. Georgalis L, Mozalevskis A, Martinez de Aragon MV, Garrido-Estepa M. Changes in the pneumococcal disease-related hospitalizations in Spain after the replacement of 7-valent by 13-valent conjugate vaccine. Eur J Clin Microbiol Infect Dis. 2017;36(3):575-83.

77. Horacio AN, Silva-Costa C, Lopes E, Ramirez M, Melo-Cristino J. Conjugate vaccine serotypes persist as major causes of non-invasive pneumococcal pneumonia in Portugal despite declines in serotypes 3 and 19A (2012-2015). PLoS One. 2018;13(11):e0206912.

78. Lopez-de-Andres A, de Miguel-Diez J, Jimenez-Trujillo I, Hernandez-Barrera V, de Miguel-Yanes JM, Mendez-Bailon M, et al. Hospitalisation with community-acquired pneumonia among patients with type 2 diabetes: an observational population-based study in Spain from 2004 to 2013. BMJ Open. 2017;7(1):e013097.

79. Mangen MJ, Huijts SM, Bonten MJ, de Wit GA. The impact of community-acquired pneumonia on the health-related quality-of-life in elderly. BMC Infect Dis. 2017;17(1):208.

80. Mangen MJ, Rozenbaum MH, Huijts SM, van Werkhoven CH, Postma DF, Atwood M, et al. Cost-effectiveness of adult pneumococcal conjugate vaccination in the Netherlands. The European respiratory journal. 2015;46(5):1407-16.

81. Menendez R, Espana PP, Perez-Trallero E, Uranga A, Mendez R, Cilloniz C, et al. The burden of PCV13 serotypes in hospitalized pneumococcal pneumonia in Spain using a novel urinary antigen detection test. CAPA study. Vaccine. 2017;35(39):5264-70.

82. Nair H, Watts AT, Williams LJ, Omer SB, Simpson CR, Willocks LJ, et al. Pneumonia hospitalizations in Scotland following the introduction of pneumococcal conjugate vaccination in young children. BMC Infect Dis. 2016;16:390.

83. Nickler M, Schaffner D, Christ-Crain M, Ottiger M, Thomann R, Hoess C, et al. Prospective evaluation of biomarkers for prediction of quality of life in community-acquired pneumonia. Clinical chemistry and laboratory medicine. 2016;54(11):1831-46.

84. Okasha O, Rinta-Kokko H, Palmu AA, Ruokokoski E, Jokinen J, Nuorti JP. Population-level impact of infant 10-valent pneumococcal conjugate vaccination on adult pneumonia hospitalizations in Finland. Thorax. 2018;73(3):262-9.

85. Patrzalek M, Kotowska M, Gorynski P, Albrecht P. Indirect effects of a 7 year PCV7/PCV13 mass vaccination program in children on the incidence of pneumonia among adults: a comparative study based on two Polish cities. Curr Med Res Opin. 2016;32(3):397-403.

86. Payeras A, Penaranda M, Inigo A, Garau M, Luis Perez J, Gallegos C, et al. Pneumococcal infections in elderly patients attending hospital since PCV-13 authorization in Spain. Infect Dis (Lond). 2017;49(1):71-80.

87. Personne V, Chevalier J, Buffel du Vaure C, Partouche H, Gilberg S, de Pouvourville G. CAPECO: Cost evaluation of community acquired pneumonia managed in primary care. Vaccine. 2016;34(19):2275-80.

88. Quan TP, Fawcett NJ, Wrightson JM, Finney J, Wyllie D, Jeffery K, et al. Increasing burden of community-acquired pneumonia leading to hospitalisation, 1998-2014. Thorax. 2016;71(6):535-42.

89. Rodrigo C, Bewick T, Sheppard C, Greenwood S, McKeever TM, Trotter CL, et al. Impact of infant 13-valent pneumococcal conjugate vaccine on serotypes in adult pneumonia. The European respiratory journal. 2015;45(6):1632-41.

90. Schmedt N, Heuer OD, Hackl D, Sato R, Theilacker C. Burden of community-acquired pneumonia, predisposing factors and health-care related costs in patients with cancer. BMC health services research. 2019;19(1):30.

91. Thorrington D, Andrews N, Stowe J, Miller E, van Hoek AJ. Elucidating the impact of the pneumococcal conjugate vaccine programme on pneumonia, sepsis and otitis media hospital admissions in England using a composite control. BMC medicine. 2018;16(1):13.

92. van Deursen AMM, Schurink-Van't Klooster TM, Man WH, van de Kassteele J, van Gageldonk-Lafeber AB, Bruijning-Verhagen P, et al. Impact of infant pneumococcal conjugate vaccination on community acquired pneumonia hospitalization in all ages in the Netherlands. Vaccine. 2017;35(51):7107-13.

93. Vila-Corcoles A, Ochoa-Gondar O, de Diego C, Satue E, Aragon M, Vila-Rovira A, et al. Evaluating clinical effectiveness of 13-valent pneumococcal conjugate vaccination against pneumonia among middle-aged and ageing adults in Catalonia: results from the EPIVAC cohort study. BMC Infect Dis. 2018;18(1):196.

94. Vissink CE, Huijts SM, de Wit GA, Bonten MJ, Mangen MJ. Hospitalization costs for community-acquired pneumonia in Dutch elderly: an observational study. BMC Infect Dis. 2016;16:466.

95. Wagenvoort GH, Sanders EA, de Melker HE, van der Ende A, Vlaminckx BJ, Knol MJ. Long-term mortality after IPD and bacteremic versus non-bacteremic pneumococcal pneumonia. Vaccine. 2017;35(14):1749-57.

96. Chalmers JD, Campling J, Dicker A, Woodhead M, Madhava H. A systematic review of the burden of vaccine preventable pneumococcal disease in UK adults. BMC Pulm Med. 2016;16(1):77.

97. Bonten MJ, Huijts SM, Bolkenbaas M, Webber C, Patterson S, Gault S, et al. Polysaccharide conjugate vaccine against pneumococcal pneumonia in adults. N Engl J Med. 2015;372(12):1114-25.

98. Campling J, Jones D, Chalmers JD, Jiang Q, Vyse A, Madhava H, et al. The impact of certain underlying co-morbidities on the risk of developing hospitalised pneumonia in England. Pneumonia (Nathan). 2019;11:4.

99. KCE. Use of pneumococcal vaccines in the elderly: an economic evaluation. 2016. Contract No.: KCE report 274

100. Naucler P, Henriques-Normark B, Hedlund J, Galanis I, Granath F, Ortqvist A. The changing epidemiology of community-acquired pneumonia: nationwide register-based study in Sweden. J Intern Med. 2019.

101. Pick H, Daniel P, Rodrigo C, Bewick T, Ashton D, Lawrence H, et al. Pneumococcal serotype trends, surveillance and risk factors in UK adult pneumonia, 2013-18. Thorax. 2019.

102. Pick HJ, Bolton CE, Lim WS, McKeever TM. Patient-reported outcome measures in the recovery of adults hospitalised with community-acquired pneumonia: a systematic review. The European respiratory journal. 2019;53(3).

103. RIVM. Pneumokokkenziekte in Nederland Achtergronddocument voor de Gezondheidsraad. 2017.

104. Vestjens SMT, Wagenvoort GHJ, Grutters JC, Meek B, Aldenkamp AF, Vlaminckx BJM, et al. Changes in pathogens and pneumococcal serotypes causing community-acquired pneumonia in The Netherlands. Vaccine. 2017;35(33):4112-8.

**Respiratory Syncytial Virus:**

105. Ambrosioni JB, P-O; Wagner, G; Mamin, A; Kaiser, L. Epidemiology of viral respiratory infections in a tertiary care centre in the era of molecular diagnosis, Geneva, Switzerland, 2011-2012. Clinical microbiology and infection : the official publication of the European Society of Clinical Microbiology and Infectious Diseases. 2014;20(9):O578-84.

106. Antalis EO, Zacharoula; Kottaridi, Christine; Kossyvakis, Athanasios; Spathis, Aris; Magkana, Maria; Katsouli, Aikaterini; Tsagris, Vassileios; Papaevangelou, Vassiliki; Mentis, Andreas; Tsiodras, Sotirios. Mixed viral infections of the respiratory tract; an epidemiological study during consecutive winter seasons. Journal of medical virology. 2018;90(4):663-70.

107. Antón AM, M A; Torner, N; Isanta, R; Camps, M; Martínez, A; Domínguez, A; Jané, M; Jiménez de Anta, M T; Pumarola, T. Virological surveillance of influenza and other respiratory viruses during six consecutive seasons from 2006 to 2012 in Catalonia, Spain. Clinical microbiology and infection : the official publication of the European Society of Clinical Microbiology and Infectious Diseases. 2016;22(6):564.e1-9.

108. Avetisyan GM, Jonas; Sparrelid, Elda; Ljungman, Per. Respiratory syncytial virus infection in recipients of allogeneic stem-cell transplantation: a retrospective study of the incidence, clinical features, and outcome. Transplantation. 2009;88(10):1222-6.

109. Bednarska KH-S, E; Kondratiuk, K; Brydak, L B. Evaluation of the Activity of Influenza and Influenza-Like Viruses in the Epidemic Season 2013/2014. Advances in experimental medicine and biology. 2015;857:1-7.

110. Bednarska KH-S, E; Kondratiuk, K; Brydak, L B. Antigenic Drift of A/H3N2/Virus and Circulation of Influenza-Like Viruses During the 2014/2015 Influenza Season in Poland. Advances in experimental medicine and biology. 2016;905:33-8.

111. Branche AR, Falsey AR. Respiratory syncytial virus infection in ageing adults: an under-recognized problem. Drugs & aging. 2015;32(4):261-9.

112. Broberg EK, Waris M, Johansen K, Snacken R, Penttinen P, Network EIS. Seasonality and geographical spread of respiratory syncytial virus epidemics in 15 European countries, 2010 to 2016. Eurosurveillance. 2018;23(5).

113. Coughtrie ALM, Denise E; Anderson, Rebecca; Begum, Nelupha; Cleary, David W; Faust, Saul N; Jefferies, Johanna M; Kraaijeveld, Alex R; Moore, Michael V; Mullee, Mark A; Roderick, Paul J; Tuck, Andrew; Whittaker, Robert N; Yuen, Ho Ming; Doncaster, C Patrick; Clarke, Stuart C. Ecology and diversity in upper respiratory tract microbial population structures from a cross-sectional community swabbing study. Journal of medical microbiology. 2018;67(8):1096-108.

114. Czarkowski MPH-S, Ewelina; Staszewska, Ewa; Bednarska, Karolina; Kondratiuk, Katarzyna; Brydak, Lidia B. Influenza in Poland in 2011-2012 and in 2011/2012 and 2012/2013 epidemic seasons. Przeglad epidemiologiczny. 2014;68(3):455-63, 559-65.

115. ECDC. Workshop on burden of RSV disease in Europe 2015 [Available from: <https://ecdc.europa.eu/sites/portal/files/media/en/press/events/Documents/Meeting%20report%20ECDC%20RSV%20surv%20and%20burden%20of%20disease%20workshop%2023-24%20Nov.pdf>.

116. Falsey ARM, Janet E; Beran, Jiri; van Essen, Gerrit A; Duval, Xavier; Esen, Meral; Galtier, Florence; Gervais, Pierre; Hwang, Shinn-Jang; Kremsner, Peter; Launay, Odile; Leroux-Roels, Geert; McNeil, Shelly A; Nowakowski, Andrzej; Richardus, Jan Hendrik; Ruiz-Palacios, Guillermo; St Rose, Suzanne; Devaster, Jeanne-Marie; Oostvogels, Lidia; Durviaux, Serge; Taylor, Sylvia. Respiratory syncytial virus and other respiratory viral infections in ageing adults with moderate to severe influenza-like illness. The Journal of infectious diseases. 2014;209(12):1873-81.

117. Fleming DMT, Robert J; Lustig, Roger L; Schuck-Paim, Cynthia; Haguinet, François; Webb, David J; Logie, John; Matias, Gonçalo; Taylor, Sylvia. Modelling estimates of the burden of Respiratory Syncytial virus infection in adults and the elderly in the United Kingdom2015 2015-Oct-23. 443 p.

118. Gaunt ERH, H; McIntyre, C; Templeton, K E; Simmonds, P. Disease burden of the most commonly detected respiratory viruses in hospitalized patients calculated using the disability adjusted life year (DALY) model. Journal of clinical virology : the official publication of the Pan American Society for Clinical Virology. 2011;52(3):215-21.

119. Gimferrer LC, Magda; Codina, María Gema; Martín, María Del Carmen; Fuentes, Francisco; Esperalba, Juliana; Bruguera, Andreu; Vilca, Luz María; Armadans, Lluís; Pumarola, Tomàs; Antón, Andrés. Molecular epidemiology and molecular characterization of respiratory syncytial viruses at a tertiary care university hospital in Catalonia (Spain) during the 2013-2014 season. Journal of clinical virology : the official publication of the Pan American Society for Clinical Virology. 2015;66:27-32.

120. Goka EV, Pamela; Mutton, Kenneth; Klapper, Paul. Influenza A viruses dual and multiple infections with other respiratory viruses and risk of hospitalisation and mortality. Influenza and other respiratory viruses. 2013;7(6):1079-87.

121. Green HKE, J; Galiano, M; Watson, J M; Pebody, R G. Critical care surveillance: insights into the impact of the 2010/11 influenza season relative to the 2009/10 pandemic season in England2013 2013-Jun-06.

122. Hardelid PP, R; Andrews, N. Mortality caused by influenza and respiratory syncytial virus by age group in England and Wales 1999-2010. Influenza and other respiratory viruses. 2013;7(1):35-45.

123. Harvala HC, AnnaSara; Axelsson, Sarah; Brytting, Maria. Evaluation of the national laboratory-based surveillance system for respiratory syncytial virus in Sweden, 2015-2016. Journal of clinical virology : the official publication of the Pan American Society for Clinical Virology. 2018;104:11-5.

124. Hughes HEM, R; Hughes, T C; Locker, T E; Pebody, R; Green, H K; Ellis, J; Smith, G E; Elliot, A J. Emergency department syndromic surveillance providing early warning of seasonal respiratory activity in England. Epidemiology and infection. 2016;144(5):1052-64.

125. Hui DSR, Giovanni A; Johnston, Sebastian L. SARS, MERS and other Viral Lung Infections. Sheffield UK: European Respiratory Society; 2016 2016-06-01.

126. Jeannoël ML, G; Rasigade, J P; Lina, B; Morfin, F; Casalegno, Jean Sebastien. Microorganisms associated with respiratory syncytial virus pneumonia in the adult population. European journal of clinical microbiology & infectious diseases : official publication of the European Society of Clinical Microbiology. 2019;38(1):157-60.

127. Kestler MM, P; Mateos, M; Adrados, D; Bouza, E. Respiratory syncytial virus burden among adults during flu season: an underestimated pathology. The Journal of hospital infection. 2018;100(4):463-8.

128. Launay O, Thalhammer F, Harrer T, Cheret A, De La Rosa G, Sander I, et al. Respiratory syncytial virus (RSV) disease in hospitalised adults in Europe: comparison of retrospective patient chart reviews of adults= 65 (= 65y) and. Eur Respiratory Soc; 2018.

129. Lehners NT, Julia; Prifert, Christiane; Wedde, Marianne; Puthenparambil, Joe; Weissbrich, Benedikt; Biere, Barbara; Schweiger, Brunhilde; Egerer, Gerlinde; Schnitzler, Paul. Long-Term Shedding of Influenza Virus, Parainfluenza Virus, Respiratory Syncytial Virus and Nosocomial Epidemiology in Patients with Hematological Disorders. PloS one. 2016;11(2):e0148258.

130. Ljungström LRJ, G; Claesson, B E B; Andersson, R; Enroth, H. Respiratory viral infections are underdiagnosed in patients with suspected sepsis. European journal of clinical microbiology & infectious diseases : official publication of the European Society of Clinical Microbiology. 2017;36(10):1767-76.

131. Loubet PL, N; Valette, M; Foulongne, V; Krivine, A; Houhou, N; Lagathu, G; Rogez, S; Alain, S; Duval, X; Galtier, F; Postil, D; Tattevin, P; Vanhems, P; Carrat, F; Lina, B; Launay, O; FLUVAC Study Group,. Clinical characteristics and outcome of respiratory syncytial virus infection among adults hospitalized with influenza-like illness in France. Clinical microbiology and infection : the official publication of the European Society of Clinical Microbiology and Infectious Diseases. 2017;23(4):253-9.

132. Morbey RAE, A J; Harcourt, S; Smith, S; de Lusignan, S; Pebody, R; Yeates, A; Zambon, M; Smith, G E. Estimating the burden on general practitioner services in England from increases in respiratory disease associated with seasonal respiratory pathogen activity. Epidemiology and infection. 2018;146(11):1389-96.

133. Morbey RAH, S; Pebody, R; Zambon, M; Hutchison, J; Rutter, J; Thomas, H; Smith, G E; Elliot, A J. The burden of seasonal respiratory infections on a national telehealth service in England. Epidemiology and infection. 2017;145(9):1922-32.

134. Nicoli EJT, Caroline L; Turner, Katherine M E; Colijn, Caroline; Waight, Pauline; Miller, Elizabeth. Influenza and RSV make a modest contribution to invasive pneumococcal disease incidence in the UK. The Journal of infection. 2013;66(6):512-20.

135. Plymoth AR-O, M; Zweygberg-Wirgart, B; Sundin, C G; Ploner, A; Nyren, O; Linde, A. Self-sampling for analysis of respiratory viruses in a large-scale epidemiological study in Sweden2015 2015-Mar-19.

136. Public Health England. Surveillance of influenza and other respiratory viruses in the UK - Winter 2018 to 2019 2019 [Available from: <https://assets.publishing.service.gov.uk/government/uploads/system/uploads/attachment_data/file/807472/Surveillance_of_influenza_and_other_respiratory_viruses_in_the_UK_2018_to_2019-FINAL.pdf>.

137. Ramaekers KK, Els; Rector, Annabel; Borremans, Annie; Beuselinck, Kurt; Lagrou, Katrien; Van Ranst, Marc. Prevalence and seasonality of six respiratory viruses during five consecutive epidemic seasons in Belgium. Journal of clinical virology : the official publication of the Pan American Society for Clinical Virology. 2017;94:72-8.

138. Rózsa M, Szalai B, Hercegh É, Bán E, Molnár Z. The Hungarian influenza surveillance associated retrospective comparative analysis of data of respiratory syncytial virus surveillance between 2013-2016 flu seasons, in the light of the influenza morbidity data. Health Science. 2017;LXI.(III).

139. Salter AL, Bairbre Ni; Crowley, Brendan. Molecular epidemiology of human respiratory syncytial virus subgroups A and B identified in adults with hematological malignancy attending an Irish hospital between 2004 and 2009. Journal of medical virology. 2011;83(2):337-47.

140. Sastre PR, Tamara; Schildgen, Oliver; Schildgen, Verena; Vela, Carmen; Rueda, Paloma. Seroprevalence of human respiratory syncytial virus and human metapneumovirus in healthy population analyzed by recombinant fusion protein-based enzyme linked immunosorbent assay. Virology journal. 2012;9:130.

141. Sendi PE, Adrian; Dangel, Marc; Frei, Reno; Tschudin-Sutter, Sarah; Widmer, Andreas F. Respiratory Syncytial Virus Infection Control Challenges with a Novel Polymerase Chain Reaction Assay in a Tertiary Medical Center. Infection control and hospital epidemiology. 2017;38(11):1291-7.

142. Shi TD, Angeline; Tietjen, Anna K; Campbell, Iain; Moran, Emily; Li, Xue; Campbell, Harry; Demont, Clarisse; Nyawanda, Bryan O; Chu, Helen Y; Stoszek, Sonia K; Krishnan, Anand; Openshaw, Peter; Falsey, Ann R; Nair, Harish; RESCEU Investigators ,. Global Disease Burden Estimates of Respiratory Syncytial Virus-Associated Acute Respiratory Infection in Ageing adults in 2015: A Systematic Review and Meta-Analysis. The Journal of infectious diseases. 2019.

143. Tanner HB, E; Osman, H. Respiratory viral infections during the 2009-2010 winter season in Central England, UK: incidence and patterns of multiple virus co-infections. European journal of clinical microbiology & infectious diseases : official publication of the European Society of Clinical Microbiology. 2012;31(11):3001-6.

144. Tsagarakis NJS, Anthi; Makridis, Panagiotis; Triantafyllou, Argyro; Stamoulakatou, Alexandra; Papadogeorgaki, Eleni. Age-related prevalence of common upper respiratory pathogens, based on the application of the FilmArray Respiratory panel in a tertiary hospital in Greece. Medicine. 2018;97(22):e10903.

145. Utsumi MM, Kiyoko; Quroshi, Nahid; Ashida, Nobuyuki. Types of infectious outbreaks and their impact in elderly care facilities: a review of the literature. Age and ageing. 2010;39(3):299-305.

146. van Asten LvdW, Cees; van Pelt, Wilfrid; van de Kassteele, Jan; Meijer, Adam; van der Hoek, Wim; Kretzschmar, Mirjam; Koopmans, Marion. Mortality attributable to 9 common infections: significant effect of influenza A, respiratory syncytial virus, influenza B, norovirus, and parainfluenza in elderly persons. The Journal of infectious diseases. 2012;206(5):628-39.

147. van Beek JV, Reinier H; Bruin, Jacob P; van Boxtel, Renée A J; de Lange, Marit M A; Meijer, Adam; Sanders, Elisabeth A M; Rots, Nynke Y; Luytjes, Willem. Influenza-like Illness Incidence Is Not Reduced by Influenza Vaccination in a Cohort of Ageing adults, Despite Effectively Reducing Laboratory-Confirmed Influenza Virus Infections. The Journal of infectious diseases. 2017;216(4):415-24.

148. Visseaux B, Burdet C, Voiriot G, Lescure F-X, Chougar T, Brugière O, et al. Prevalence of respiratory viruses among adults, by season, age, respiratory tract region and type of medical unit in Paris, France, from 2011 to 2016. PloS one. 2017;12(7):e0180888.

149. Vos LM, Teirlinck AC, Lozano JE, Vega T, Donker GA, Hoepelman AI, et al. Use of the moving epidemic method (MEM) to assess national surveillance data for respiratory syncytial virus (RSV) in the Netherlands, 2005 to 2017. Eurosurveillance. 2019;24(20).

150. Zhao H, Green H, Lackenby A, Donati M, Ellis J, Thompson C, et al. A new laboratory-based surveillance system (Respiratory DataMart System) for influenza and other respiratory viruses in England: results and experience from 2009 to 2012. Eurosurveillance. 2014;19(3):20680.

151. Murray CJ, Lopez AD, Organization WH. The global burden of disease: a comprehensive assessment of mortality and disability from diseases, injuries, and risk factors in 1990 and projected to 2020: summary. 1996.

152. Sanchez-Luna M, Elola FJ, Fernandez-Perez C, Bernal JL, Lopez-Pineda A. Trends in respiratory syncytial virus bronchiolitis hospitalizations in children less than 1 year: 2004–2012. Current medical research and opinion. 2016;32(4):693-8.

**Staphylococcus aureus:**

153. Asgeirsson H, Gudlaugsson O, Kristinsson KG, Heiddal S, Kristjansson M. Staphylococcus aureus bacteraemia in Iceland, 1995-2008: changing incidence and mortality. 2011;17(4):513-8.

154. Thorlacius-Ussing L, Sandholdt H, Larsen AR, Petersen A, Benfield T. Age-Dependent Increase in Incidence of Staphylococcus aureus Bacteremia, Denmark, 2008-2015. 2019;25(5).

155. Bonnet I, Millon B, Meugnier H, Vandenesch F, Maurin M, Pavese P, et al. High prevalence of spa type t571 among methicillin-susceptible Staphylococcus aureus from bacteremic patients in a French University Hospital. 2018;13(10):e0204977.

156. Abelenda Alonso G, Corbacho Loarte MD, Núñez Ramos R, Cervero Jiménez M, Jusdado Ruiz-Capillas JJ. Staphylococcus aureus bacteremia in a secondary level Spanish hospital: clinical implications of high vancomycin MIC. 2018;31(4):353-62.

157. Alfandari S, Cabaret P, Nguyen S, Descamps D, Vachée A, Cattoen C, et al. Evaluating the management of 493 patients presenting with bacteremia in 23 northern French hospitals2016 2016-Jun %J Medecine et maladies infectieuses. 194-9 p.

158. ECDC. External quality assessment of laboratory performance - European Antimicrobial Resistance Surveillance Network (EARS-Net), 2017. Stockholm: ECDC; 2018.

159. Asgeirsson H, Kristjansson M, Kristinsson KG, Gudlaugsson O. Staphylococcus aureus bacteraemia--Nationwide assessment of treatment adequacy and outcome. 2011;62(5):339-46.

160. Bassetti M, Peghin M, Trecarichi EM, Carnelutti A, Righi E, Del Giacomo P, et al. Characteristics of Staphylococcus aureus Bacteraemia and Predictors of Early and Late Mortality. 2017;12(2):e0170236.

161. Bassetti M, Righi E, Del Giacomo P, Sartor A, Ansaldi F, Trucchi C, et al. Predictors of Mortality with Staphylococcus aureus Bacteremia in Elderly Adults. 2018;66(7):1284-9.

162. Bassetti M, Trecarichi EM, Mesini A, Spanu T, Giacobbe DR, Rossi M, et al. Risk factors and mortality of healthcare-associated and community-acquired Staphylococcus aureus bacteraemia. 2012;18(9):862-9.

163. Beeston CJ, Gupta R, Chadwick PR, Young RJ. Methicillin-resistant Staphylococcus aureus bacteraemia and mortality in a teaching hospital. 2009;28(6):585-90.

164. Berger J, Diab-Elschahawi M, Blacky A, Pernicka E, Spertini V, Assadian O, et al. A matched prospective cohort study on Staphylococcus aureus and Escherichia coli bloodstream infections: extended perspectives beyond resistance. 2010;38(10):839-45.

165. Blomfeldt A, Eskesen AN, Aamot HV, Leegaard TM, Bjørnholt JV. Population-based epidemiology of Staphylococcus aureus bloodstream infection: clonal complex 30 genotype is associated with mortality. 2016;35(5):803-13.

166. Bouiller K, Gbaguidi-Haore H, Hocquet D, Cholley P, Bertrand X, Chirouze C. Clonal complex 398 methicillin-susceptible Staphylococcus aureus bloodstream infections are associated with high mortality. 2016;22(5):451-5.

167. Bourneton O, Mutel T, Heranney D, Hernandez C, Lavigne T, Waller J, et al. [Incidence of hospital-acquired and community-acquired bloodstream infections in the University of Strasbourg Hospitals, France, between 2005 and 2007]2010 2010-Feb %J Pathologie-biologie. 29-34 p.

168. Brady M, Oza A, Cunney R, Burns K. Attributable mortality of hospital-acquired bloodstream infections in Ireland. 2017;96(1):35-41.

169. Buetti N, Marschall J, Atkinson A, Kronenberg A, Swiss Centre for Antibiotic Resistance National Bloodstream Infection Surveillance in Switzerland 2008-2014: Different Patterns and Trends for University and Community Hospitals2016 2016-09 %J Infection control and hospital epidemiology. 1060-7 p.

170. Wiese L, Mejer N, Schønheyder HC, Westh H, Jensen AG, Larsen AR, et al. A nationwide study of comorbidity and risk of reinfection after Staphylococcus aureus bacteraemia. 2013;67(3):199-205.

171. Braquet P, Alla F, Cornu C, Goehringer F, Piroth L, Chirouze C, et al. Factors associated with 12 week case-fatality in Staphylococcus aureus bacteraemia: a prospective cohort study. 2016;22(11):948.e1-.e7.

172. Corrah TW, Enoch DA, Aliyu SH, Lever AM. Bacteraemia and subsequent vertebral osteomyelitis: a retrospective review of 125 patients. 2011;104(3):201-7.

173. Cuervo G, Camoez M, Shaw E, Dominguez MÁ, Gasch O, Padilla B, et al. Methicillin-resistant Staphylococcus aureus (MRSA) catheter-related bacteraemia in haemodialysis patients. 2015;15:484.

174. Cuervo G, Gasch O, Shaw E, Camoez M, Domínguez MÁ, Padilla B, et al. Clinical characteristics, treatment and outcomes of MRSA bacteraemia in the elderly2016 2016-Mar %J The Journal of infection. 309-16 p.

175. Cuny C, Layer F, Werner G, Harmsen D, Daniels-Haardt I, Jurke A, et al. State-wide surveillance of antibiotic resistance patterns and spa types of methicillin-resistant Staphylococcus aureus from blood cultures in North Rhine-Westphalia, 2011-2013. 2015;21(8):750-7.

176. De Rosa FG, Corcione S, Motta I, Petrolo A, Filippini C, Pagani N, et al. Risk factors for mortality in patients with Staphylococcus aureus bloodstream infection. 2016;28(3):187-90.

177. Deptuła A, Trejnowska E, Dubiel G, Wanke-Rytt M, Deptuła M, Hryniewicz W. Healthcare associated bloodstream infections in Polish hospitals: prevalence, epidemiology and microbiology-summary data from the ECDC Point Prevalence Survey of Healthcare Associated Infections 2012-2015. 2018;37(3):565-70.

178. Diekema DJ, Pfaller MA, Shortridge D, Zervos M, Jones RN. Twenty-Year Trends in Antimicrobial Susceptibilities Among Staphylococcus aureus From the SENTRY Antimicrobial Surveillance Program. 2019;6:S47-S53.

179. Djuric O, Jovanovic S, Stosovic B, Tosic T, Jovanovic M, Nartey N, et al. Differences in MRSA prevalence and resistance patterns in a tertiary center before and after joining an international program for surveillance of antimicrobial resistance2017 2017-Jun-01 %J Acta microbiologica et immunologica Hungarica. 165-77 p.

180. Eskesen AN, Belle MA, Blomfeldt A. Predictors of one-year all-cause mortality and infection-related mortality in patients with Staphylococcus aureus bacteraemia. 2018;50(10):743-8.

181. Finkelstein R, Agmon Y, Braun E, Kassis I, Sprecher H, Raz A, et al. Incidence and risk factors for endocarditis among patients with health care-associated Staphylococcus aureus bacteraemia. 2012;44(12):934-40.

182. Forsblom E, Kakriainen A, Ruotsalainen E, Järvinen A. Comparison of patient characteristics, clinical management, infectious specialist consultation, and outcome in men and women with methicillin-sensitive Staphylococcus aureus bacteremia: a propensity-score adjusted retrospective study2018 2018-Dec %J Infection. 837-45 p.

183. Forsblom E, Kakriainen A, Ruotsalainen E, Järvinen A. Methicillin-sensitive Staphylococcus aureus bacteremia in aged patients: the importance of formal infectious specialist consultation. 2018;9(3):355-63.

184. Forsblom E, Ruotsalainen E, Mölkänen T, Ollgren J, Lyytikäinen O, Järvinen A. Predisposing factors, disease progression and outcome in 430 prospectively followed patients of healthcare- and community-associated Staphylococcus aureus bacteraemia. 2011;78(2):102-7.

185. Forstner C, Dungl C, Tobudic S, Mitteregger D, Lagler H, Burgmann H. Predictors of clinical and microbiological treatment failure in patients with methicillin-resistant Staphylococcus aureus (MRSA) bacteraemia: a retrospective cohort study in a region with low MRSA prevalence. 2013;19(7):E291-7.

186. Fysaraki M, Samonis G, Valachis A, Daphnis E, Karageorgopoulos DE, Falagas ME, et al. Incidence, clinical, microbiological features and outcome of bloodstream infections in patients undergoing hemodialysis. 2013;10(12):1632-8.

187. Gasch O, Ayats J, Angeles Dominguez M, Tubau F, Liñares J, Peña C, et al. Epidemiology of methicillin-resistant Staphylococcus aureus (MRSA) bloodstream infection: secular trends over 19 years at a university hospital2011 2011-Sep %J Medicine. 319-27 p.

188. Gasch O, Camoez M, Domínguez MA, Padilla B, Pintado V, Almirante B, et al. Predictive factors for early mortality among patients with methicillin-resistant Staphylococcus aureus bacteraemia. 2013;68(6):1423-30.

189. Gasch O, Camoez M, Dominguez MA, Padilla B, Pintado V, Almirante B, et al. Predictive factors for mortality in patients with methicillin-resistant Staphylococcus aureus bloodstream infection: impact on outcome of host, microorganism and therapy. 2013;19(11):1049-57.

190. Geffers C, Gastmeier P. Nosocomial infections and multidrug-resistant organisms in Germany: epidemiological data from KISS (the Hospital Infection Surveillance System). 2011;108(6):87-93.

191. Gibbons CL, van Bunnik BAD, Blatchford O, Robertson C, Porphyre T, Imrie L, et al. Not just a matter of size: a hospital-level risk factor analysis of MRSA bacteraemia in Scotland. 2016;16:222.

192. Gotland N, Uhre ML, Mejer N, Skov R, Petersen A, Larsen AR, et al. Long-term mortality and causes of death associated with Staphylococcus aureus bacteremia. A matched cohort study. 2016;73(4):346-57.

193. Gubbels S, Nielsen J, Voldstedlund M, Kristensen B, Schønheyder HC, Vandenbroucke-Grauls CMJE, et al. Utilization of blood cultures in Danish hospitals: a population-based descriptive analysis. 2015;21(4):344.e13-21.

194. Guembe M, Pérez-Granda MJ, Capdevila JA, Barberán J, Pinilla B, Martín-Rabadán P, et al. Nationwide study on peripheral-venous-catheter-associated-bloodstream infections in internal medicine departments. 2017;97(3):260-6.

195. Hagstrand Aldman M, Skovby A, I Påhlman L. Penicillin-susceptible Staphylococcus aureus: susceptibility testing, resistance rates and outcome of infection. 2017;49(6):454-60.

196. Hansen M-LU, Gotland N, Mejer N, Petersen A, Larsen AR, Benfield T, et al. Diabetes increases the risk of disease and death due to Staphylococcus aureus bacteremia. A matched case-control and cohort study. 2017;49(9):689-97.

197. Hanses F, Spaeth C, Ehrenstein BP, Linde H-J, Schölmerich J, Salzberger B. Risk factors associated with long-term prognosis of patients with Staphylococcus aureus bacteremia. 2010;38(6):465-70.

198. Hernández C, Fehér C, Soriano A, Marco F, Almela M, Cobos-Trigueros N, et al. Clinical characteristics and outcome of elderly patients with community-onset bacteremia. 2015;70(2):135-43.

199. Heudorf U, Mischler D, Bobyk D, Bornhofen B, Maiwald M, Merbs R, et al. [MRSA bloodstream infections according to the German obligation for notification - data and experience of the MDRO Network Rhine-Main, Germany, 2011]2014 2014-Jun %J Gesundheitswesen (Bundesverband der Arzte des Offentlichen Gesundheitsdienstes (Germany)). 385-91 p.

200. Heudorf U, Otto U, Gottschalk R. [MRSA bloodstream infections in hospitals in Frankfurt/Main, Germany, 2010 : Results of the mandatory notification and suggestions for improvement]2011 2011-Sep %J Bundesgesundheitsblatt, Gesundheitsforschung, Gesundheitsschutz. 1126-34 p.

201. Hoenigl M, Wagner J, Raggam RB, Prueller F, Prattes J, Eigl S, et al. Characteristics of hospital-acquired and community-onset blood stream infections, South-East Austria. 2014;9(8):e104702.

202. Jacobsson G, Nasic S. Long-term outcome of invasive Staphylococcus aureus infections. 2012;44(5):350-4.

203. Jogenfors A, Stark L, Svefors J, Löfgren S, Malmvall B-E, Matussek A. A recommendation to perform a blood culture before the administration of intravenous antibiotics increased the detection of Staphylococcus aureus bacteremia. 2014;33(5):789-95.

204. Jokinen E, Laine J, Huttunen R, Lyytikäinen O, Vuento R, Vuopio J, et al. Trends in incidence and resistance patterns of Staphylococcus aureus bacteremia. 2018;50(1):52-8.

205. Jokinen E, Laine J, Huttunen R, Rahikka P, Huhtala H, Vuento R, et al. Comparison of outcome and clinical characteristics of bacteremia caused by methicillin-resistant, penicillin-resistant and penicillin-susceptible Staphylococcus aureus strains2017 2017-Jul %J Infectious diseases (London, England). 493-500 p.

206. Joost I, Kaasch A, Pausch C, Peyerl-Hoffmann G, Schneider C, Voll RE, et al. Staphylococcus aureus bacteremia in patients with rheumatoid arthritis - Data from the prospective INSTINCT cohort. 2017;74(6):575-84.

207. Joseph JP, Meddows TR, Webster DP, Newton JD, Myerson SG, Prendergast B, et al. Prioritizing echocardiography in Staphylococcus aureus bacteraemia. 2013;68(2):444-9.

208. Kaasch AJ, Barlow G, Edgeworth JD, Fowler VG, Hellmich M, Hopkins S, et al. Staphylococcus aureus bloodstream infection: a pooled analysis of five prospective, observational studies. 2014;68(3):242-51.

209. Kızılarslanoğlu MC, Sancak B, Yağcı S, Hasçelik G, Unal S. [Evaluation of methicillin-resistant Staphylococcus aureus bacteremia and comparison of prognosis according to vancomycin MIC values: experience of the last ten years]2013 2013-Apr %J Mikrobiyoloji bulteni. 199-210 p.

210. Lalueza A, Morales-Cartagena A, Chaves F, San Juan R, Laureiro J, Lora-Tamayo J, et al. Risk factors for metastatic osteoarticular infections after a long follow-up of patients with Staphylococcus aureus bacteraemia. 2015;21(11):1010.e1-5.

211. Lamagni TL, Potz N, Powell D, Pebody R, Wilson J, Duckworth G. Mortality in patients with meticillin-resistant Staphylococcus aureus bacteraemia, England 2004-2005. 2011;77(1):16-20.

212. Laupland KB, Lyytikäinen O, Søgaard M, Kennedy KJ, Knudsen JD, Ostergaard C, et al. The changing epidemiology of Staphylococcus aureus bloodstream infection: a multinational population-based surveillance study. 2013;19(5):465-71.

213. Le Moing V, Alla F, Doco-Lecompte T, Delahaye F, Piroth L, Chirouze C, et al. Staphylococcus aureus Bloodstream Infection and Endocarditis--A Prospective Cohort Study2015 2015 %J PloS one. e0127385 p.

214. Lessa FC, Mu Y, Davies J, Murray M, Lillie M, Pearson A, et al. Comparison of incidence of bloodstream infection with methicillin-resistant Staphylococcus aureus between England and United States, 2006-20072010 2010-Oct-15 %J Clinical infectious diseases : an official publication of the Infectious Diseases Society of America. 925-8 p.

215. Lewis T, Chaudhry R, Nightingale P, Lambert P, Das I. Methicillin-resistant Staphylococcus aureus bacteremia: epidemiology, outcome, and laboratory characteristics in a tertiary referral center in the UK. 2011;15(2):e131-5.

216. López Del Pino P, Guerrero Espejo A. Incidence and mortality of osteomielitis in Spain according to the basic minimum data set. 2019.

217. Luzzaro F, Ortisi G, Larosa M, Drago M, Brigante G, Gesu G. Prevalence and epidemiology of microbial pathogens causing bloodstream infections: results of the OASIS multicenter study. 2011;69(4):363-9.

218. Mehl A, Åsvold BO, Lydersen S, Paulsen J, Solligård E, Damås JK, et al. Burden of bloodstream infection in an area of Mid-Norway 2002-2013: a prospective population-based observational study. 2017;17(1):205.

219. Mejer N, Westh H, Schønheyder HC, Jensen AG, Larsen AR, Skov R, et al. Stable incidence and continued improvement in short term mortality of Staphylococcus aureus bacteraemia between 1995 and 2008. 2012;12:260.

220. Melzer M, Welch C. Thirty-day mortality in UK patients with community-onset and hospital-acquired meticillin-susceptible Staphylococcus aureus bacteraemia2013 2013-Jun %J The Journal of hospital infection. 143-50 p.

221. Morales-Cartagena A, Fernández-Ruiz M, Lalueza A, Lora-Tamayo J, San Juan R, López-Medrano F, et al. Impact on mortality of adherence to evidence-based interventions in patients with catheter-related bloodstream infection due to methicillin-sensitive Staphylococcus aureus. 2018;50(11):837-46.

222. Morris AK, Russell CD. Enhanced surveillance of Staphylococcus aureus bacteraemia to identify targets for infection prevention. 2016;93(2):169-74.

223. Murdoch F, Danial J, Morris AK, Czarniak E, Bishop JL, Glass E, et al. The Scottish enhanced Staphylococcus aureus bacteraemia surveillance programme: the first 18 months of data in adults. 2017;97(2):133-9.

224. N'Guyen Y, Baumard S, Vernet-Garnier V, Batalla AS, de Champs C, Jaussaud R, et al. Coagulase-negative Staphylococcus bacteraemia accounts for one third of Staphylococcus bacteraemia in a French university hospital. 2012;44(2):79-85.

225. Nielsen LH, Jensen-Fangel S, Benfield T, Skov R, Jespersen B, Larsen AR, et al. Risk and prognosis of Staphylococcus aureus bacteremia among individuals with and without end-stage renal disease: a Danish, population-based cohort study. 2015;15:6.

226. Nielsen SL, Lassen AT, Gradel KO, Jensen TG, Kolmos HJ, Hallas J, et al. Bacteremia is associated with excess long-term mortality: A 12-year population-based cohort study. 2015;70(2):111-26.

227. Nielsen SL, Pedersen C, Jensen TG, Gradel KO, Kolmos HJ, Lassen AT. Decreasing incidence rates of bacteremia: a 9-year population-based study. 2014;69(1):51-9.

228. NNSR. AZ ORSZÁGOS EPIDEMIOLÓGIAI KÖZPONT TÁJÉKOZTATÓJA A NEMZETI NOSOCOMIALIS SURVEILLANCE RENDSZER 2017. ÉVI EREDMÉNYEIRŐL. 2017.

229. Oestergaard LB, Schmiegelow MD, Bruun NE, Skov RL, Petersen A, Andersen PS, et al. The associations between socioeconomic status and risk of Staphylococcus aureus bacteremia and subsequent endocarditis - a Danish nationwide cohort study. 2017;17(1):589.

230. Paulsen J, Mehl A, Askim Å, Solligård E, Åsvold BO, Damås JK. Epidemiology and outcome of Staphylococcus aureus bloodstream infection and sepsis in a Norwegian county 1996-2011: an observational study. 2015;15:116.

231. Pleşca C-E, Luca MC, Luca AS, Constantinescu S, Iancu LS. ETIOLOGY AND RESISTANCE PROFILE OF ISOLATED STRAINS FROM SEVERE SYSTEMIC INFECTIONS IN PATIENTS WITH IMMUNODEPRESSION--EXPERIENCE OF THE IASI INFECTIOUS DISEASES HOSPITAL 2011-2014. 2015;119(4):1141-6.

232. Rasmussen G, Monecke S, Brus O, Ehricht R, Söderquist B. Long term molecular epidemiology of methicillin-susceptible Staphylococcus aureus bacteremia isolates in Sweden. 2014;9(12):e114276.

233. Reunes S, Rombaut V, Vogelaers D, Brusselaers N, Lizy C, Cankurtaran M, et al. Risk factors and mortality for nosocomial bloodstream infections in elderly patients2011 2011-Oct %J European journal of internal medicine. e39-44 p.

234. Rieg S, Peyerl-Hoffmann G, de With K, Theilacker C, Wagner D, Hübner J, et al. Mortality of S. aureus bacteremia and infectious diseases specialist consultation--a study of 521 patients in Germany. 2009;59(4):232-9.

235. Rodríguez-Baño J, López-Prieto MD, Portillo MM, Retamar P, Natera C, Nuño E, et al. Epidemiology and clinical features of community-acquired, healthcare-associated and nosocomial bloodstream infections in tertiary-care and community hospitals. 2010;16(9):1408-13.

236. Roth JA, Widmer AF, Tschudin-Sutter S, Dangel M, Frei R, Battegay M, et al. The Model for End-stage Liver Disease (MELD) as a predictor of short-term mortality in Staphylococcus aureus bloodstream infection: A single-centre observational study. 2017;12(4):e0175669.

237. Santini M, Kutlesa M, Pangercic A, Barsic B. The importance of pathogens in sepsis: Staphylococcus aureus story. 2010;42(3):172-6.

238. Schweickert B, Noll I, Feig M, Claus H, Krause G, Velasco E, et al. MRSA-surveillance in Germany: data from the Antibiotic Resistance Surveillance System (ARS) and the mandatory surveillance of MRSA in blood. 2012;31(8):1855-65.

239. Skogberg K, Lyytikäinen O, Ollgren J, Nuorti JP, Ruutu P. Population-based burden of bloodstream infections in Finland. 2012;18(6):E170-6.

240. Smit J, Adelborg K, Thomsen RW, Søgaard M, Schønheyder HC. Chronic heart failure and mortality in patients with community-acquired Staphylococcus aureus bacteremia: a population-based cohort study. 2016;16:227.

241. Smit J, López-Cortés LE, Kaasch AJ, Søgaard M, Thomsen RW, Schønheyder HC, et al. Gender differences in the outcome of community-acquired Staphylococcus aureus bacteraemia: a historical population-based cohort study. 2017;23(1):27-32.

242. Smit J, Thomsen RW, Schønheyder HC, Nielsen H, Frøslev T, Søgaard M. Outcome of Community-Acquired Staphylococcus aureus Bacteraemia in Patients with Diabetes: A Historical Population-Based Cohort Study. 2016;11(4):e0153766.

243. Stammler Jaliff B, Dahl-Knudsen J, Petersen A, Skov R, Benfield T. Outcome and reinfection after Staphylococcus aureus bacteraemia in individuals with and without HIV-1 infection: a case-control study. 2014;4(4):e004075.

244. Ştefan G, Stancu S, Căpuşă C, Ailioaie OR, Mircescu G. Catheter-related infections in chronic hemodialysis: a clinical and economic perspective2013 2013-Jun %J International urology and nephrology. 817-23 p.

245. van Cleef BAGL, Kluytmans JAJW, van Benthem BHB, Haenen A, Monen J, Daniels-Haardt I, et al. Cross border comparison of MRSA bacteraemia between The Netherlands and North Rhine-Westphalia (Germany): a cross-sectional study2012 2012 %J PloS one. e42787 p.

246. van Cleef BAGL, van Benthem BHB, Haenen APJ, Bosch T, Monen J, Kluytmans JAJW. Low incidence of livestock-associated methicillin-resistant Staphylococcus aureus bacteraemia in The Netherlands in 2009. 2013;8(8):e73096.

247. Velasco C, López-Cortés LE, Caballero FJ, Lepe JA, de Cueto M, Molina J, et al. Clinical and molecular epidemiology of meticillin-resistant Staphylococcus aureus causing bacteraemia in Southern Spain. 2012;81(4):257-63.

248. Walter J, Haller S, Blank H-P, Eckmanns T, Abu Sin M, Hermes J. Incidence of invasive meticillin-resistant Staphylococcus aureus infections in Germany, 2010 to 2014. 2015;20(46).

249. Widmer AF, Lakatos B, Frei R. Strict infection control leads to low incidence of methicillin-resistant Staphylococcus aureus bloodstream infection over 20 years. 2015;36(6):702-9.

250. Wilson J, Elgohari S, Livermore DM, Cookson B, Johnson A, Lamagni T, et al. Trends among pathogens reported as causing bacteraemia in England, 2004-2008. 2011;17(3):451-8.

251. Wilson J, Guy R, Elgohari S, Sheridan E, Davies J, Lamagni T, et al. Trends in sources of meticillin-resistant Staphylococcus aureus (MRSA) bacteraemia: data from the national mandatory surveillance of MRSA bacteraemia in England, 2006-2009. 2011;79(3):211-7.

252. Wolkewitz M, Frank U, Philips G, Schumacher M, Davey P, BURDEN Study Group. Mortality associated with in-hospital bacteraemia caused by Staphylococcus aureus: a multistate analysis with follow-up beyond hospital discharge. 2011;66(2):381-6.

253. WHO. Central Asian and Eastern European Surveillance of Antimicrobial Resistance. Annual report 2017. Copenhagen: WHO Regional Office for Europe; 2018.
